# Supplementary material for: Homeotic compartment curvature and tension control spatiotemporal folding dynamics
Source: Nat Commun. 2023 Feb 3;14:594. doi: 10.1038/s41467-023-36305-6 (PMC9898526; doi:10.1038/s41467-023-36305-6)
Supplement: Supplementary file 1 — Supplementary Information [file 41467_2023_36305_MOESM1_ESM.pdf]

Supplementary information

Homeotic compartment curvature and tension control spatiotemporal folding dynamics.

Aurélien Villedieu[1,2], Lale Alpar[1,2], Isabelle Gaugue[1,2], Amina Joudat[1,2], François Graner[3], Floris Bosveld[1,2,4,*] and Yohanns Bellaïche[1,2,4,*]

1 : Institut Curie, PSL Research University, CNRS UMR 3215, INSERM U934, F-75248 Paris Cedex 05, Paris, France.

2 : Sorbonne Universités, UPMC Univ Paris 06, CNRS, CNRS UMR 3215, INSERM U934, F-75005, France.

3 : Université Paris Cité, CNRS, Matière et Systèmes Complexes, F-75006 Paris, France.

4: These authors jointly supervised this work.

Corresponding authors: floris.bosveld@curie.fr; yohanns.bellaiche@curie.fr

Contents

Supplementary Note

Supplementary Figures and legends

Supplementary Table 1.

Supplementary references

Supplementary Note

In this Supplementary Note, we describe the quantitative analysis and biophysical model that we have used to understand neck folding. Section 1 details the registration and quantification approaches to quantitatively measure neck folding and average it over multiple animals in different mutant contexts. In Section 2 we build upon these quantitative methods, the 3D characterization of cell shape and laser ablation experiments to explore the contributions of apical constriction, apical-basal shortening, cell delamination associated with apoptosis and buckling in the regulation of neck folding. In Section 3, based on the analysis of the distribution of MyoII, the curvature of the Dfd domain and mechanical stress estimation, we then provide a self-contained description of the simple physical consideration leading to the modeling of neck folding. Last, in Section 4 we provide additional considerations regarding the geometrical feedback associated with the stabilization of neck curvature, independently of the initial heterogeneity in curvature.

1. Registration and quantification of neck folding and normal deepening speed.

In order to quantify neck fold invagination and deepening speed the Ecad::3xGFP signal was imaged in the head-thorax region of a pupa by acquiring *z*-stacks covering a depth of 40 µm below the initial tissue plane. Upon *z*-stack projection, the resulting image at 14-17 hAPF was first visualized in top view in order to (i) identify the landmark macrochaetae located in the head and in the thorax necessary for spatial registration along the medial-lateral ($\text{Position}_{\text{ML}}$) and anterior-posterior axes ($\text{Position}_{\text{AP}}$) (Supplementary Fig. 1c and see also Methods section for details); and (ii) to track the apical fold front, which is located at the neck-thorax boundary. The *z*-stacks were then resliced (20 µm, region indicated by red brackets in Supplementary Fig. 1c) around the location of the apical fold front to obtain transverse views along the ML axis (Supplementary Fig. 1d, blue line defines position of apical fold front). The animal midline position was used as the axis of bilateral symmetry (dashed orange line in Supplementary Fig. 1c-f) and enabled to define coordinates along the ML axis ($\text{Position}_{\text{ML}}$). The $\text{Position}_{\text{ML}} = 0\%$ can be used as the ML position of the center of convergence of neck invagination since it corresponds to the position of the neck in the adult: its location was estimated using pictures of the back of the adult head on a transverse section (Supplementary Fig. 1e,f). The apical fold front was then tracked in time (Supplementary Fig. 1g). The depth of the apical fold front was then calculated for each $\text{Position}_{\text{ML}}$ and each time point (Supplementary Fig. 1h). Finally, for each time point, depths were averaged for $\text{Position}_{\text{ML}}$ between -250% and 250% to obtain the curve of the apical fold front deepening dynamics in each analyzed animal (Supplementary Fig. 1i), then averaged over multiple animals.

2. Analyses of the contribution of apical constriction, cell apical-basal shortening, delamination and compressive tissue flows in neck folding.

We explored known mechanisms of tissue folding to analyze whether they might explain the observed neck folding: (i) cell apical constriction concomitant with basal relaxation[1–10]; (ii) apical-basal shortening[11–14]; (iii) delamination associated with cell apoptosis[15,16]; and (iv) tissue buckling due to compressive stresses[3,17–22]. As shown in Supplementary Fig. 6, our data indicate that these mechanisms do not contribute substantially to neck folding.

(i) *Apical constriction concomitant with basal relaxation*: In folds driven by apical constriction, apical cell length reduces in a direction perpendicular to the folding axis, while basal cell length increases; thus leading to cell wedging. This is controlled by distinct apical and basal tension changes in a direction perpendicular to the fold[23]. In contrast, here neck cells did not wedge along the AP axis (i.e. perpendicular to the fold axis) between 16 hAPF and 24 hAPF, but cells reduced their basal sides more than their apical sides (Supplementary Fig. 6a-d). In addition, we found that apical and basal recoil velocities perpendicular to the fold (i.e., AP orientation) are low and similar (Supplementary Fig. 2a). Therefore, the observed cell shape changes and the apical and basal tensions indicate that neck folding is unlikely to be mediated by known apical constriction mechanisms driving folding.

(ii) *Apical-basal shortening:* The tissue apical-basal shortening around the fold front corresponded to only a very small fraction of the fold depth (Supplementary Fig. 6e,f). As lateral tension increase has been linked with apical-basal shortening[11–14], this agrees with the finding that lateral cell tension is too low to be detectable by lateral cell laser ablation (Supplementary Fig. 6g,h). Therefore, apical-basal shortening is not a main contributor to neck folding in the pupa.

(iii) *Folding driven by delamination associated with apoptosis:* In contrast to apoptosis-driven folding[15,16], here neck cell delamination was not concomitant with a local and transient apical plane bending (Supplementary Fig. 6k). Moreover, reducing apoptosis in the neck by overexpressing the anti-apoptotic *Diap1* gene using a *Dfd-GAL4* driver (*Dfd>Diap1*) did not prevent folding, and if anything, slightly increased its speed (Supplementary Fig. 6l,m). While Dfd has been reported to control segment boundary formation by promoting apoptosis in the *Drosophila* embryo folding[24], apoptosis does not appear to be a major contributor of neck folding.

(iv) *Tissue buckling*: To test the role of putative compressive flows in neck folding, we aimed at abrogating the converging head and thorax tissue flows by laser ablation prior to folding. These laser ablations disrupted the head and thorax flow of cells (Supplementary Fig. 6n), but they did not abrogate the initiation of neck invagination (Supplementary Fig. 6o). Yet, around 6 h following ablation, the deepening speed started to decrease relative to non-ablated control tissue, presumably as a long-term consequence of the tissue stretching observed in the head and thorax cells near the ablation sites (Supplementary Fig. 6n,o). We therefore tested whether the head and thorax flows are needed to maintain neck folding by performing similar ablations during folding. When head and thorax tissue flows were abrogated at 21 hAPF, neck folding proceeded normally (Supplementary Fig. 6o). These ablation experiments therefore suggest that head and thorax compressive flows are not required during the initiation and the maintenance phases of neck folding.

Collectively, these experimental findings indicate that the aforementioned mechanisms of tissue folding do not make a major contribution during neck folding.

3. Neck folding modeling and energy dissipation.

Here we provide a self-contained description of neck folding modeling, as a thin layer the movement of which is driven by Laplace force and limited by viscous dissipation.

a. Balance between Laplace and viscous forces.

The magnitude of the Laplace force, per unit length of curve, is the tension $\mathbf{T}$ multiplied by the curvature κ. Since neck folding is associated with large scale tissue flows, we have favored a viscous description of energy dissipation. Inertia being negligible with respect to viscous dissipation, we write that the sum of the Laplace force and of the normal dissipative force is zero; the normal dissipative force being the deepening speed (velocity $\mathbf{v_n}$ of the curve along the vector normal to the curve), multiplied by an unknown negative dissipative prefactor, $-\mu$. So, the prediction is that $\kappa\mathbf{T} - \mu\mathbf{v_n} = 0$ or equivalently, that $\mathbf{v_n}$ is proportional to $\kappa\mathbf{T}$ and is directed inwards. Note that this prediction $\kappa\mathbf{T} - \mu\mathbf{v_n} = 0$ is local and involves the tension independently of its cause and of the boundary conditions (whether anchored or periodic).

b. Thin layer description.

From the theoretical point of view, since the tissue thickness (~10 μm) is smaller than the neck radius (~300 μm) of curvature, the tissue can reasonably be treated as a thin layer. From the experimental point of view, the physical model presented here is a data-driven analysis using the classical equation of Laplace force, of general validity, to perform predictions. Altogether, this model of a line under tension yields correct predictions of neck folding dynamics for both control and mutant conditions in which mechanical tension is altered.

We have chosen to treat the apical and basal sides of the tissue separately based on the following experimental and theoretical considerations. We have delineated how genetic regulators, Dfd, Tollo and

Dys/Dg determine apical and basal tensions. The apical "tension times curvature" product correlates with the apical tissue velocity, which determines the apical line position versus time; while the basal "tension times curvature" product correlates with the basal velocity which determines the basal line position versus time. The apical quantities are determined with a better signal-to-noise ratio than the basal ones. Both apical and basal velocities are well and separately predicted by the model up to an unknown prefactor, so the absolute values of the velocities are not predicted. The apical and basal prefactors can differ (for instance due to differences in the basal or apical ECM), thus the difference between apical and basal velocities is not predicted. At each time, the difference between apical and basal line positions determines the tissue thickness, which is thus downstream of genetics and velocities, and varies with time. We have quantified this thickness variation with time. Last, our experimental data do not suggest applying the equation of Laplace force to the tissue as a whole to try to predict a « tissue velocity » using the product of a « tissue curvature » with a « tissue tension ». In particular, the velocities, curvatures and tensions differ on apical and basal sides, so that defining them at tissue scale might be ambiguous.

c. Negligible effect from the coverslip on the energy dissipation.

As the neck epithelium harbors an apical ECM (aECM, the cuticle), the coverslip used for live imaging is not in direct contact with the epithelium. In principle, the presence of the coverslip in contact with the aECM could indirectly modulate the friction, the adhesion between the aECM and the epithelium, as well as compress the tissue. The friction of the epithelium on the aECM could induce a force on apical cell surfaces, opposed to the velocity of cells along the coverslip. This, however, would not modify the components of the force and velocity which we observe here, which are perpendicular to the coverslip. Therefore, even if the coverslip-induced flattening changed the friction between the aECM and the epithelium, this would not explain the strong correlation between the deepening speed and the product of curvature and tension. In addition, in principle the coverslip could hinder the cell movements for instance through adhesion. There are strong indications that this is not the case: (i) all the thorax imaging published so far was performed using similar coverslip flattening, yet thorax flow and tissue deformation reproducibly occur at large scale[25,26]; (ii) before 18 hAPF, the neck cells actively move and the neck tissue deforms in plane, hence it is unlikely that cells would be strongly attached (Supplementary Movie 1); (iii) in Figs. 5d and 6j, instead of a linear relationship we would have detected a discontinuity (within experimental uncertainty in the curvature measurement, we would have detected at least a significant difference) between the attached part of the tissue, which would have zero deepening velocity, and the non-attached tissue, which is curved and free to invaginate. In summary, the strong linear correlation between the deepening speed and the product of curvature and tension (recoil velocity) can reasonably exclude that the local modulation of neck folding dynamics with coverslip flattening could be explained by the friction or the adhesion between the aECM and the epithelium in the regions flattened by the coverslip.

d. Negligible effect from the coverslip on other mechanical properties.

The flattening conditions might induce compression on the tissue. Therefore, in principle all the experiments that modulate curvature could also alter concomitantly at least another property of the tissue. In particular, the flattening we impose slightly affects the tension (Supplementary Fig. 11a,b). In principle, it could also affect the mechanical properties of the neck and surrounding tissues, especially through mechanotransduction. Here, the relative change of surface due to the flattening, estimated by comparing the initial surface length before flattening (the arc of circle) with the flattened surface width (the corresponding chord) is of order of 3.7 ± 0.5%. The shear deformation, estimated by the ratio of tissue surface displacement towards the pupa center to the flattened surface width, is of order of 10.6 ± 1.3%. This is well within linear elasticity[27], "linear" meaning here that the effects are proportional to the cause, and hence likely to remain of order of a few percent too: the modification of mechanical properties is expected to remain moderate. We note that tissue thickness at the midline measured at 18 hAPF has a large variability and is 8.6 ± 1.8 µm for flattened animals ($N = 10$) vs 7.5 ± 1 µm for non-

flattened ones ($N = 10$); hence the effect of flattening, which is to increase rather than to decrease the tissue thickness, is not significant ($p = 0.11$). In addition, what we ultimately check is that even if the values of tension, curvature or velocity change, their correlation remains and is compatible with the equation of Laplace force, and thus, with a role of curvature in tuning the spatiotemporal dynamics of neck invagination.

4. Robustness and temporal evolution of neck curvature upon flattening.

Upon medial flattening of the neck region, the curvature of the midline region gradually increases in time as the neck folds (Supplementary Fig. 11d) and the curvature increase propagates from the more lateral regions to the more medial ones (Supplementary Fig. 11e). Therefore, while the medial neck region is initially flat, over time its curvature becomes like the one observed in the absence of medial flattening. Such stabilization of the neck curvature is a property of curvature-driven movements that robustly yields a homogeneous curvature when shrinking. In fact, consider a curve in which the curved regions move faster than less curved ones. Then, for purely geometrical reasons, when this curve shrinks the curved regions shrink faster, the less curved regions shrink more slowly, and their curvature increases until it equals that of the more curved regions. Hence, the curve shape robustly becomes an arc of a circle (i.e., has a spatially homogenous curvature). Note that in principle another known mechanism could lead to curvature homogenization, namely if the dissipation coefficient μ was anisotropic as is the case when the curve moves within a crystalline lattice[28,29]. Here, because of the experimental quantitative linear agreement of the product of tension and curvature with deepening speed, such anisotropic mechanism is excluded.

Supplementary Figures and legends

Supplementary Fig. 1: Quantification of neck folding dynamics.

(**a**) Graph of the neck, head, and thorax apical cell anisotropy (± sd) at 13 hAPF (n = 40 cells from N = 2 pupae for each tissue region). ANOVA: Neck vs Head p = 1e-6, Neck vs Thorax p = 1e-6, Head vs Thorax p = 0.118.

(**b**) 3D image of Ecad::3xGFP and Dfd localizations in the dorsal neck region at 24 hAPF to illustrate the distribution of Dfd in the most lateral region of the neck region accessible with our imaging setup. The cropped version is shown in Fig. 1c.

(**c**) Top view image of the head, neck and thorax regions labelled by Ecad::3xGFP at 16 hAPF. Red and yellow circles, landmark macrochaetae of the head and thorax, respectively, used to define the coordinates along the ML ($Position_{ML}$) and AP ($Position_{AP}$) axes. Green line, neck-thorax boundary. Orange dashed line, midline position. Orange brackets at the neck-thorax interface corresponds to a 20 µm box used to track the apical fold front in transverse sections. The magenta bracket indicates the neck region.

(**d**) Transverse view at 16 hAPF of the apical fold front region labelled by Ecad::3xGFP. $Position_{ML}$ are indicated. Blue line, apical fold front. Orange dashed line, midline position.

(**e**) Transverse view of the back of the adult head. Blue dashed line: estimated flattening applied by the coverslip on the animal during imaging. Grey circle: estimated center of convergence of neck folding. Orange dashed line, axis of bilateral symmetry and distance between midline and center (approximately 200 µm). Red circles, landmark macrochaetae of the head.

(**f**) Position of apical fold front on transverse view at 16 hAPF, plotted with the estimated center of convergence of neck invagination (grey circle). Orange dashed line, axis of bilateral symmetry and distance between midline and center (approximately 200 µm). The black dashed lines indicate the approximate successive positions of points at given $Position_{ML}$ during neck folding.

(**g**) Color-coded successive positions of the apical fold front for the animal shown in (d) tracked over developmental time. Black dashed lines represent the estimated successive positions of points at given $Position_{ML}$ during neck folding. The successive positions were determined using the estimated center of convergence (see e,f).

(**h**) Graph of the depth of apical fold front as a function of $Position_{ML}$ and developmental time. White dashed lines delimit the +250% and -250% $Position_{ML}$ between which fold depth is averaged at each time point to generate the graph in (i).

(**i**) Graph of the average depth of apical fold front (between $Position_{ML}$ = -250% and 250%) of the animal in (d) versus developmental time.

Source data are provided as a Source Data file.

Scale bars, 40 µm (b), 50 µm (c-g). ANOVA, ns $p > 0.5$, **** $p < 0.00001$.

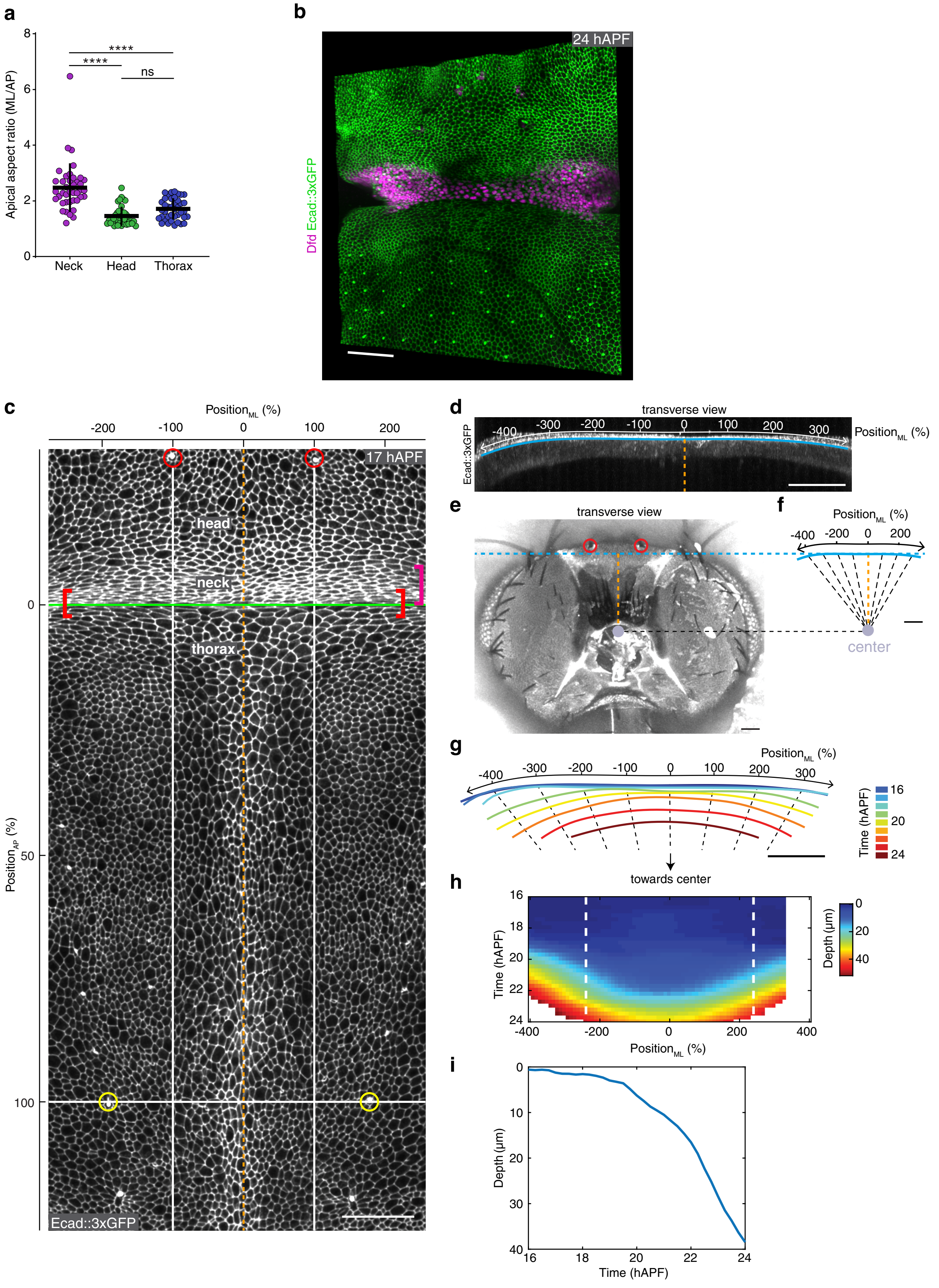
a
Apical aspect ratio (ML/AP)
****
****
ns
Neck
Head
Thorax
b
24 hAPF
Dfd Ecad::3xGFP
c
Position_ML (%)
17 hAPF
head
neck
thorax
Position_AP (%)
Ecad::3xGFP
d
transverse view
Ecad::3xGFP
Position_ML (%)
e
transverse view
f
Position_ML (%)
center
g
Position_ML (%)
towards center
Time (hAPF)
h
Time (hAPF)
Depth (µm)
Position_ML (%)
i
Depth (µm)
Time (hAPF)

Supplementary Fig. 2: Actin organization, measurements of apical and basal tensions and neck apical folding.

(**a**) Graph of the apical (top panels) and basal (bottom panels) initial recoil velocities (mean ± sd) after ablations measured along the AP and ML axes at 22 hAPF in head (apical N = 18 pupae, basal N = 10 pupae), neck (apical and basal N = 18 pupae) and thorax (apical N = 19 pupae, basal N = 16 pupae) regions to estimate AP and ML tissue tension. Welch test: Head apical AP vs ML $p = 0.028$, Neck apical AP vs ML p = 6.29e-11, Thorax apical AP vs ML p = 4.7e-4, Head basal AP vs ML p = 6.63e-3, Neck basal AP vs ML p = 5.07e-08, Thorax basal AP vs ML $p = 0.475$. Circular ablations were used to measure recoil simultaneously along both the AP and the ML axes. As AP tension is negligible all further reported measurements of recoil velocities were performed in a rectangular ROI (b) to analyze ML tissue recoil velocities.

(**b**) Top view images of utrABD::GFP actin labelling in the apical and basal neck tissue at 22 hAPF prior to ML ablation ($t = 0$ s) and after ablation ($t = 7$ s) in the indicated ROIs (red dashed boxes). Yellow arrows indicate tissue recoil used to measure tissue recoil velocity between $t = 1$ s and $t = 7$ s to estimate tissue tension.

(**c**) Graph of the apical versus basal ML recoil velocity after laser ablation at different developmental times. Each point is the mean value (± sd) among 127 animals for a given color-coded developmental time. The correlation between the two recoil velocities is indicated ($R^2 = 0.95$).

(**d**) Top view image of the neck region labelled by Ecad::3xGPF at 21 hAPF indicating the two regions (medial, dark blue and lateral, light blue) where apical and basal recoil velocities were measured after laser ablation (see e and f). Dashed line, midline position. Yellow line, neck-thorax boundary.

(**e**) Graph of the ML apical initial recoil (mean ± sd, averaged with a 2 h sliding window) velocities measured upon ablation in the medial (dark blue, N = 127 pupae) and lateral (light blue, N = 45 pupae) neck regions as a function of developmental time. Horizontal boxes: p-values of Welch tests performed between pupae ablated medially and laterally at successive time points (white $p > 0.05$, striped $p < 0.05$, solid $p < 0.01$).

(**f**) Graph of the ML basal initial recoil (mean ± sd, averaged with a 2 h sliding window) velocities measured upon ablation in the medial (dark blue, N = 127 pupae) and lateral (light blue, N = 45 pupae) neck regions as a function of developmental time. Horizontal boxes: p-values of Welch tests performed between pupae ablated medially or laterally at successive time points (white $p > 0.05$, striped $p < 0.05$, solid $p < 0.01$).

(**g**) Transverse view time-lapse images of the neck region at 18, 21 and 24 hAPF in wt and *Dfd-GAL4* control conditions, as well as in $Dfd{>}Rok^{RNAi}$, $Dfd{>}MyoII^{RNAi}$, $Dfd{>}Mbs^{RNAi}$ representative animals. Tissues are labelled with Ecad::3xGFP. Colored lines outline the position of the apical fold front at successive time points (blue 18 hAPF, yellow 21 hAPF, red 24 hAPF). Note that in the $Dfd{>}Mbs^{RNAi}$ the tissue has moved out of the field view at 24 hAPF and the apical fold front line cannot be shown.

(**h**) Graph of the apical neck depth (blue, mean ± sem) in pupae mock injected with H_2O (blue, N = 6 pupae) and 50 mM Y-27632 (red, N = 8 pupae) as a function of developmental time. Apical neck depth is defined relative to the initial position of the AJ labeled by Ecad::3xGFP at the onset of imaging. The region in grey corresponds to the period during which the injection was performed, and time-lapse imaging was stopped (see Methods for details). Horizontal boxes: p-values of Welch tests performed between pupae injected with H_2O or Y-27632 at successive time points (white $p > 0.05$, striped $p < 0.05$, solid $p < 0.01$).

Source data are provided as a Source Data file.

Scale bars, 20 µm (b), 50 µm (d,g). Welch test, ns $p > 0.5$, * $p < 0.05$, ** $p < 0.01$, *** $p < 0.001$.

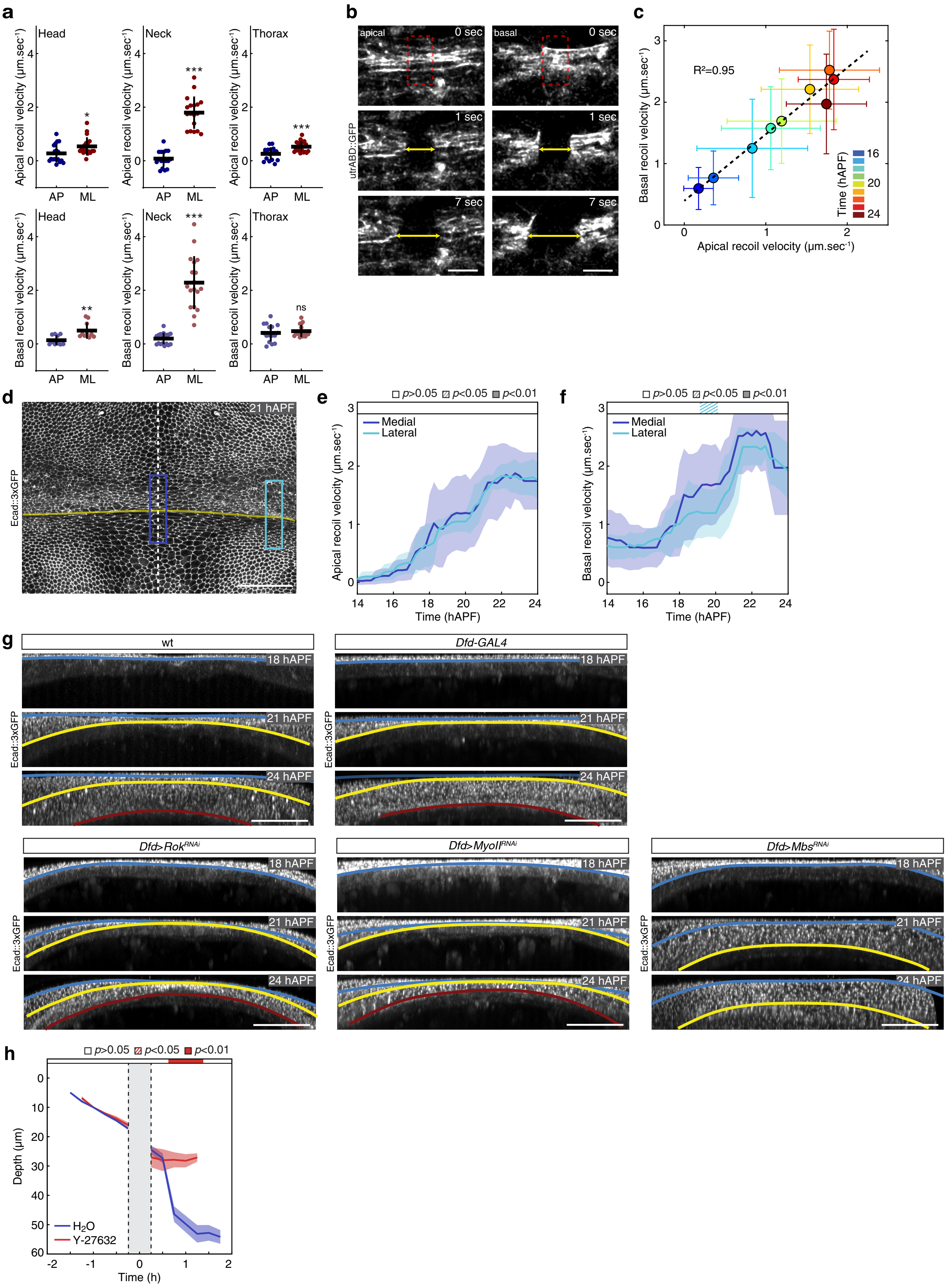

a
Head
Neck
Thorax
Apical recoil velocity (µm.sec⁻¹)
Basal recoil velocity (µm.sec⁻¹)
AP
ML
*
***
**
ns
b
apical
basal
0 sec
1 sec
7 sec
utrABD::GFP
c
R²=0.95
Basal recoil velocity (µm.sec⁻¹)
Apical recoil velocity (µm.sec⁻¹)
Time (hAPF)
16
20
24
d
21 hAPF
Ecad::3xGFP
e
f
p>0.05
p<0.05
p<0.01
Medial
Lateral
Time (hAPF)
g
wt
Dfd-GAL4
Dfd>Rok^RNAi
Dfd>MyoII^RNAi
Dfd>Mbs^RNAi
18 hAPF
21 hAPF
24 hAPF
h
Depth (µm)
Time (h)
H₂O
Y-27632

Supplementary Fig. 3: *Dfd*, *Tollo* and *Dys*/*Dg* regulate the assembly of neck apical and basal actomyosin structures and neck folding dynamics.

(**a**) Dfd (grey left, magenta right) and basal F-Actin (grey middle, green right) localization in the neck region at 22 hAPF in control *Dfd>GAL4* and *Dfd>Dfd^{RNAi}* mutant tissues. The regions outlined in orange on the left panels are shown next to it with a higher gain. At high gain, some Dfd signal can be detected in the neck in *Dfd>Dfd^{RNAi}* mutant tissues and the neck region appears enlarged in this mutant condition. In particular, the most anterior domain (blue brackets) of the Dfd expression domain does not seem to be affected. Magenta brackets, neck region.

(**b**) Transverse view time-lapse images of the neck region at 18, 21 and 24 hAPF in wt and *Dfd-GAL4* control conditions, as well as in a *Dfd>Dfd^{RNAi}* representative animal. Tissues are labelled with Ecad::3xGFP. Colored lines outline the position of the apical fold front at successive time points (blue 18 hAPF, yellow 21 hAPF, red 24 hAPF).

(**c**) Graph of the neck apical cell anisotropy (± sem) for *Dfd-GAL4* (n = 45 cells from N = 3 pupae for 13 and 14.5 hAPF, n = 105 cells from N = 7 pupae for 16, 18, and 20 hAPF), *Dfd>Dfd^{RNAi}* (n = 30 cells from N = 2 pupae for 13 and 14.5 hAPF, n = 105 cells from N = 7 pupae for 16, 18, and 20 hAPF), *Dfd>Dg^{RNAi}* (n = 45 cells from N = 3 pupae for 13 and 14.5 hAPF, n = 120 cells from N = 8 pupae for 16, 18, and 20 hAPF) and *Dfd>Tollo^{RNAi}* (n = 45 cells from N = 3 pupae for 13 and 14.5 hAPF, n = 105 cells from N = 7 pupae for 16, 18, and 20 hAPF) between 13 and 20 hAPF.

(**d**) Basal F-actin localization (top) and segmentation of the F-actin fibers color-coded by orientation (bottom) at 21 hAPF in control *Dfd>GAL4*, *Dfd>Dfd^{RNAi}* and *Dys* mutant tissues. Segmented F-actin fibers are colored according to their orientation, ranging from 0° (along ML axis) to 90° (along the AP axis). While F-Actin fiber orientation is affected in *Dfd>Dfd^{RNAi}* and *Dys* mutant tissues (e), the phalloidin signal level is similar to the ones observed in control *Dfd>GAL4* mutant tissues. Blue and magenta brackets indicate the thorax and neck regions respectively where F-actin fiber orientation was analyzed.

(**e**) Graph of the F-actin fibers orientation relative the ML axis (mean ± sd) in control *Dfd-GAL4* (N = 9 pupae), *Dfd>Dfd^{RNAi}* (N = 9 pupae) and *Dys* (N = 7 pupae) mutant tissues quantified in the neck (left) or the thorax (right) regions at 21 hAPF. Each F-actin fiber orientation is measured between 0 and 90° relative to the ML axis (i.e., 0° corresponding to a fiber parallel to the ML axis). Each dot is the fiber orientation averaged for one animal. Welch test: Neck *Dfd>Dfd^{RNAi}* p = 2.82e-07, *Dys* p = 1.19e-3, Thorax *Dfd>Dfd^{RNAi}* p = 0.097, *Dys* p = 3.09e-3.

Source data are provided as a Source Data file.

Scale bars, 20 µm (a,d), 50 µm (b). Welch test, ns $p > 0.5$, ** $p < 0.05$, *** $p < 0.001$.

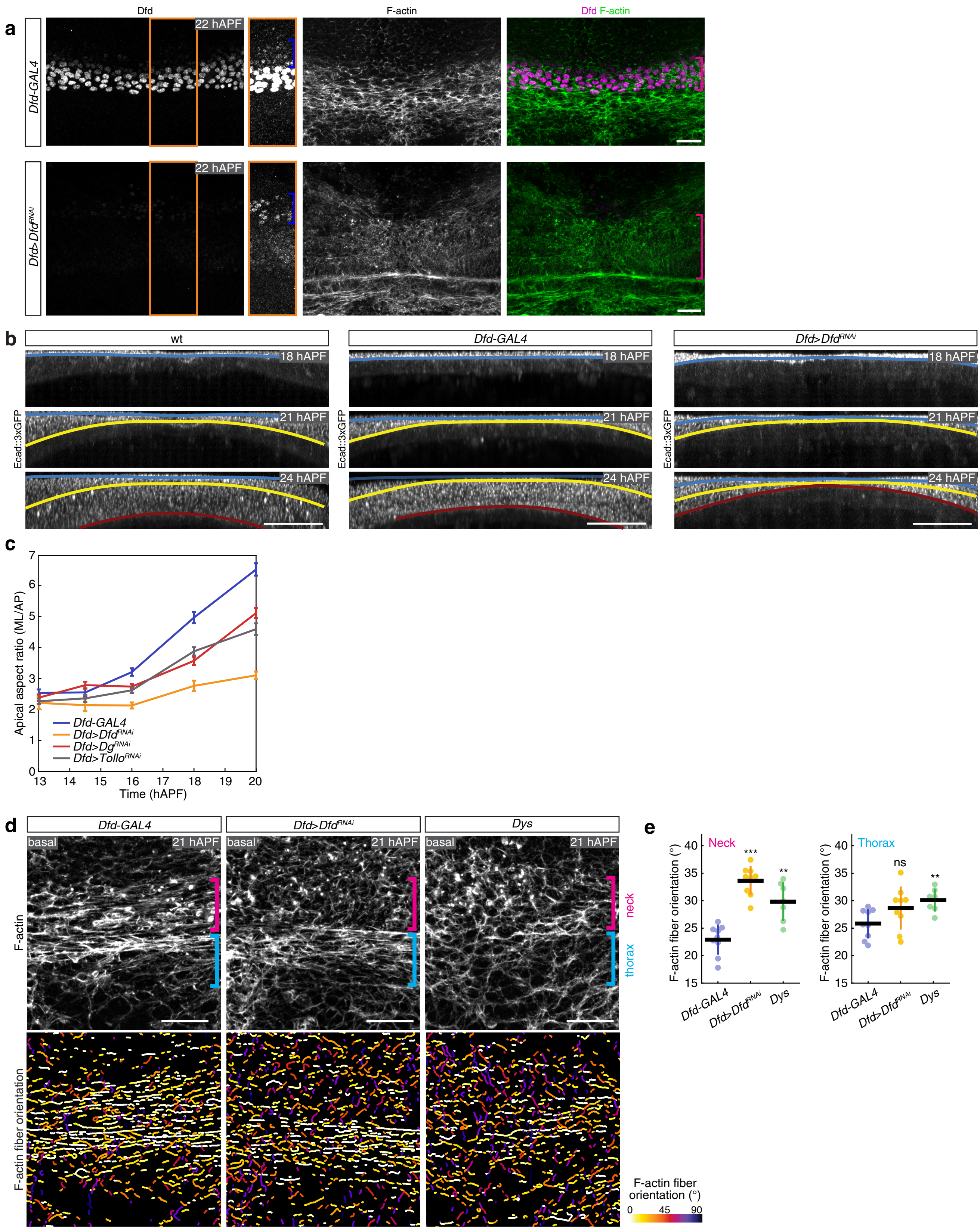
a
Dfd
F-actin
Dfd F-actin
Dfd-GAL4
Dfd>Dfd^RNAi
22 hAPF
b
wt
Dfd-GAL4
Dfd>Dfd^RNAi
Ecad::3xGFP
18 hAPF
21 hAPF
24 hAPF
c
Apical aspect ratio (ML/AP)
Time (hAPF)
Dfd-GAL4
Dfd>Dfd^RNAi
Dfd>Dg^RNAi
Dfd>Tollo^RNAi
d
Dfd-GAL4
Dfd>Dfd^RNAi
Dys
basal
21 hAPF
F-actin
neck
thorax
F-actin fiber orientation
F-actin fiber orientation (°)
0 45 90
e
Neck
Thorax
F-actin fiber orientation (°)
***
**
ns
**
Dfd-GAL4
Dfd>Dfd^RNAi
Dys

Supplementary Fig. 4: Tollo and Dys/Dg mutant neck folding dynamics.

(**a**) Sagittal view of Tollo::YFP (grey left, green right) and F-actin (magenta right) distributions in the neck region at 21 hAPF.

(**b**) Sagittal view of Dg::GFP (grey left, green right) and F-actin (magenta right) distributions in the neck region at 21 hAPF.

(**c**) Sagittal view of Dys::GFP (grey left, green right) and F-actin (magenta right) distributions in the neck region at 21 hAPF.

(**d**) Transverse view time-lapse images of the neck region at 18, 21 and 24 hAPF in wt and *Dfd-GAL4* control conditions, as well as in *Dfd>Tollo*^{RNAi}, *Tollo*, *Dys*, *Dg* and *Dfd>Dg*^{RNAi} representative animals. Tissues are labelled with Ecad::3xGFP, except for *Dg* which is labelled with ubi-Ecad::GFP. The ubi:Ecad::GFP transgene also promotes Ecad::GFP expression in circulating cells that are present below the tissue. Colored lines outline the position of the apical fold front at successive time points (blue 18 hAPF, yellow 21 hAPF, red 24 hAPF).

(**e**) Graph of apical neck depth (mean ± sem) in wt ($N = 10$ pupae) and *Tollo* ($N = 7$ pupae) mutant tissues as a function of developmental time. Horizontal box: *p*-values of Welch tests performed between the wt control and *Tollo* mutant condition at successive time points (white $p > 0.05$, striped $p < 0.05$, solid $p < 0.01$).

(**f**) Graph of apical neck depth (mean ± sem) in *Dfd-GAL4* ($N = 9$ pupae) and *Dfd>Dg*^{RNAi} ($N = 9$ pupae) mutant tissues as a function of developmental time. Horizontal box: *p*-values of Welch tests performed between the *Dfd-GAL4* control and *Dfd>Dg*^{RNAi} mutant condition at successive time points (white $p > 0.05$, striped $p < 0.05$, solid $p < 0.01$).

Source data are provided as a Source Data file.

Scale bars, 10 µm (a-c), 50 µm (d).

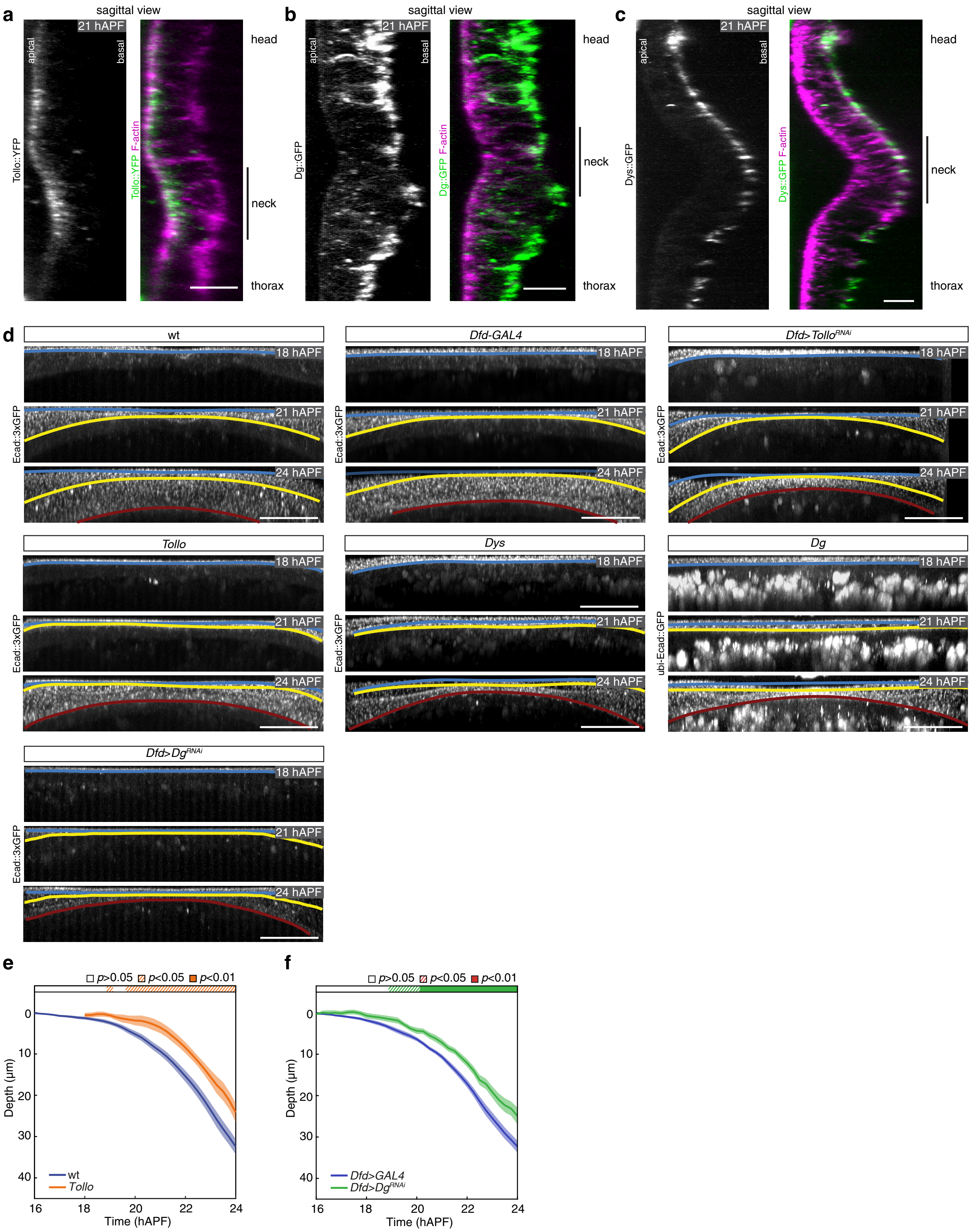
a
sagittal view
21 hAPF
apical
basal
Tollo::YFP
Tollo::YFP F-actin
head
neck
thorax
b
sagittal view
21 hAPF
apical
basal
Dg::GFP
Dg::GFP F-actin
head
neck
thorax
c
sagittal view
21 hAPF
apical
basal
Dys::GFP
Dys::GFP F-actin
head
neck
thorax
d
wt
Dfd-GAL4
Dfd>Tollo^RNAi
Tollo
Dys
Dg
Dfd>Dg^RNAi
18 hAPF
21 hAPF
24 hAPF
Ecad::3xGFP
ubi-Ecad::GFP
e
p>0.05 p<0.05 p<0.01
Depth (μm)
Time (hAPF)
wt
Tollo
f
p>0.05 p<0.05 p<0.01
Depth (μm)
Time (hAPF)
Dfd>GAL4
Dfd>Dg^RNAi

Supplementary Fig. 5: Interplays between Tollo and Dg function during neck folding.

(**a**) Graph of apical neck depth (mean ± sem) in *Dfd-GAl4* control (N = 9 pupae), *Dfd>Dfd*^{RNAi} (N = 8 pupae), *Dys* (N = 12 pupae), *Dfd>Tollo*^{RNAi} (N = 11 pupae) and *Dys,Dfd>Tollo*^{RNAi} (N = 8 pupae) mutant tissues as a function of developmental time. Horizontal boxes: p-values of Welch tests performed between the *Dfd-GAl4* control and *Dfd>Dfd*^{RNAi} (orange) or *Dfd>Tollo*^{RNAi} (dark grey) or *Dys,Dfd>Tollo*^{RNAi} (purple) as well as between wt control and *Dys* (green) at successive time points (white $p > 0.05$, striped $p < 0.05$, solid $p < 0.01$).

(**b**) Basal Dg::GFP (grey left, green right) and F-actin (grey middle, green right) distributions in control *Dfd>GAL4* (top) and *Dfd>Tollo*^{RNAi} (bottom) mutant tissues at 21 hAPF. Magenta brackets, neck region.

(**c**) Apical Tollo::YFP (grey top, green bottom) in *Dg*^{RNAi} clones, marked by Caax::BFP accumulation (magenta bottom right) at 21 hAPF. Yellow dashed line, outlines of *Dg*^{RNAi} clones. A close up of the region outlined in orange is shown next to the panel. Yellow line, neck-thorax boundary.

Source data are provided as a Source Data file.

Scale bars, 20 µm.

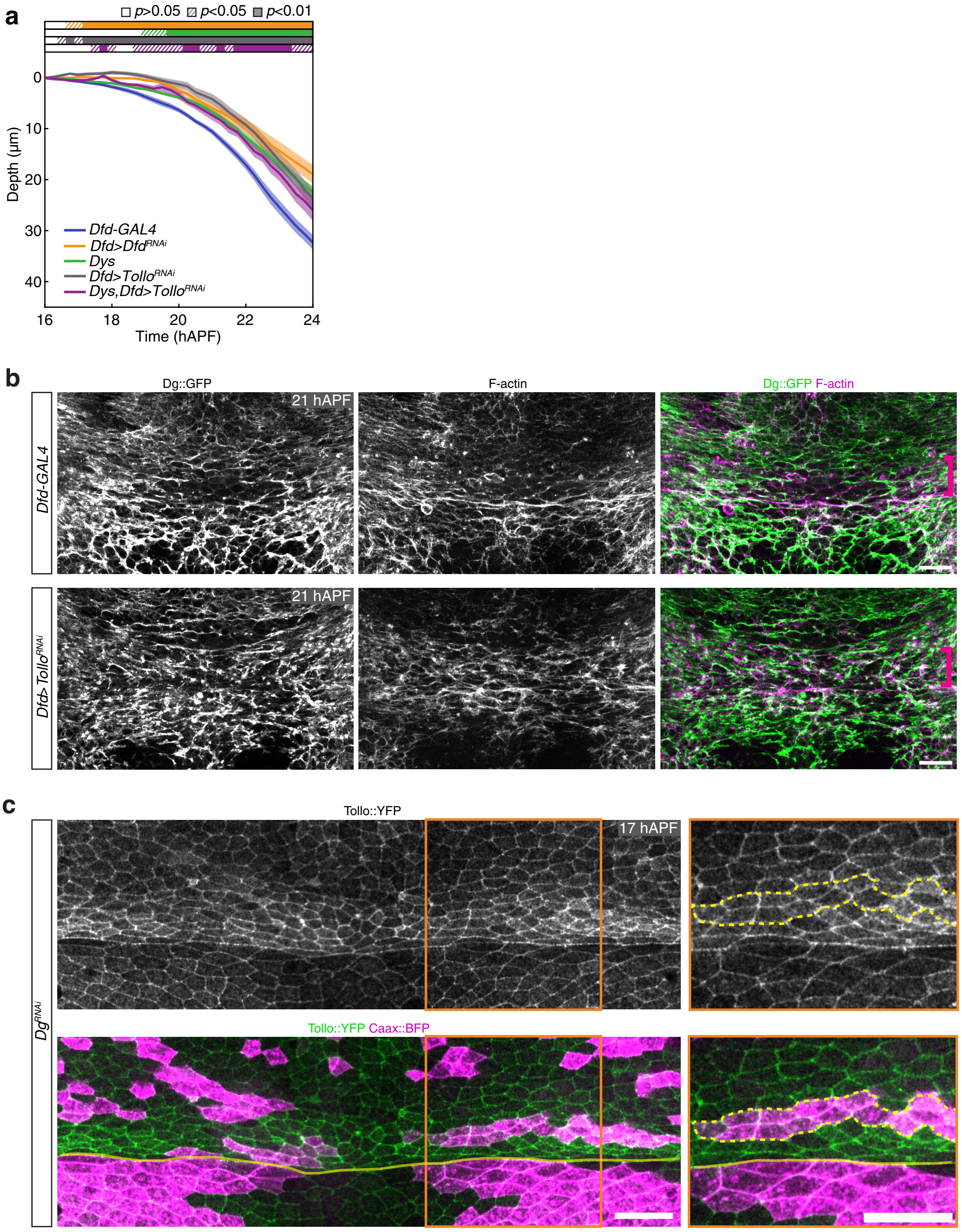

a
p>0.05 p<0.05 p<0.01
Depth (μm)
0
10
20
30
40
16
18
20
22
24
Time (hAPF)
Dfd-GAL4
Dfd>Dfd^RNAi
Dys
Dfd>Tollo^RNAi
Dys,Dfd>Tollo^RNAi
b
Dg::GFP
F-actin
Dg::GFP F-actin
21 hAPF
Dfd-GAL4
Dfd>Tollo^RNAi
c
Tollo::YFP
17 hAPF
Tollo::YFP Caax::BFP
Dg^RNAi

Supplementary Fig. 6: Analyses of the contribution of apical constriction, cell apical-basal shortening, delamination and compressive tissue flows in neck folding (see also Supplementary Note).

(**a**) Sagittal view images of the PH::GFP labelled cell membranes in the neck region at 16 and 24 hAPF. Arrows indicate neck-thorax interface.

(**b**) 3D views of a row of cells in the head, neck and thorax regions centered around the midline at 16 and 24 hAPF.

(**c**) Graphs of the apical (left, Welch test $p = 0.26$) and basal (middle, Welch test $p = 8e\text{-}5$) cell length (mean ± sd) along the AP axis (l_{AP}) at 16 hAPF and 24 hAPF and of the apical-basal cell shape changes along the AP axis between 16 and 24 hAPF (right, *t*-test $p = 3.2e\text{-}4$) measured using the PH::GFP marker. The apical-basal cell shape change along the AP axis was measured as the ratio between the apical and basal cell length variation along this axis. For each time point 75 cells were measured from $N = 3$ pupae.

(**d**) Graphs of the apical (left, Welch test $p = 0.133$) and basal (middle, Welch test $p = 0.833$) cell length (mean ± sd) along the ML axis (l_{ML}) at 16 hAPF and 22 hAPF and of the apical-basal cell shape changes along the ML axis between 16 and 24 hAPF (right, *t*-test $p = 0.148$) measured using the PH::GFP marker. The apical-basal cell shape change along the ML axis was measured as the ratio between the apical and basal cell length variation along this axis. For each time point 75 cells were measured from $N = 3$ pupae. *t*-test, ns $p > 0.05$.

(**e**) Transverse view kymograph of the neck region labelled by Ecad:3xGFP from 16 to 24 hAPF. Blue and pink dashed lines indicate the successive apical (blue) and basal (pink) fold front positions of the neck tissue, respectively.

(**f**) Graph of apical neck depth (blue, mean ± sem) and of cumulative tissue height reduction (red, mean ± sem) as a function of developmental time ($N = 10$ pupae). Apical neck depth is defined relative to the initial position of the AJ labeled by Ecad::3xGFP. Tissue height is defined by the difference between the apical and basal Ecad::3xGFP signal (see e).

(**g**) Images of apical-basal Ecad::3xGFP distribution on a transverse view in the neck region at 21 hAPF prior to ablation and 30 sec after ablation of the lateral region (red dashed box). Arrowheads, positions of Ecad::3xGFP labelled tissue prior to and after ablation. Since this laser power is sufficient to ablate the actomyosin network of the tissue at a deeper position to trigger tissue recoil, we can safely consider that this regime is sufficient to ablate the lateral cortex to estimate recoil velocity.

(**h**) Graph of the out of plane ($N = 11$ pupae) recoil velocity (mean ± sd) upon lateral tissue ablation.

(**i**) Graph of the ML apical initial recoil velocities in the medial neck region (mean ± sd) without (control) ($N = 30$ pupae) and after a preceding basal tissue ablation ($N = 13$ pupae). Welch test $p = 0.33$. Ablations were performed at 22 ± 1 hAPF.

(**j**) Graph of the ML basal initial recoil velocities in the medial neck region (mean ± sd) without (control) ($N = 13$ pupae) and after a preceding apical tissue ablation ($N = 30$ pupae). Welch test $p = 0.38$. Ablations were performed at 22 ± 1 hAPF.

(**k**) Top (top panels) and lateral view (bottom panels) time-lapse images of Ecad::3xGFP and MyoII::3xmKate2 in a neck cell which is delaminating. Yellow arrows, contours of the delaminating cell. Note the absence of tissue bending in the apical plane during delamination (bottom panels).

(**l**) Graph of delamination events (mean ± sd) from 14 hAPF to 21 hAPF in control tissue (*Dfd-GAL4*, $N = 8$ pupae) and tissue overexpressing Diap1 (*Dfd>Diap1*, $N = 8$ pupae). Delamination events were quantified in an equivalent neck region centered around the midline in all animals. Welch test $p = 1e\text{-}5$.

(**m**) Graph of apical neck depth (mean ± sem) in control *Dfd-GAL4* ($N = 9$ pupae) and *Dfd>Diap1* ($N = 12$ pupae) tissues as a function of developmental time. Horizontal box: *p*-values of Welch tests performed between the *Dfd-GAL4* control and *Dfd>Diap1* mutant condition at successive time points (white $p > 0.05$, striped $p < 0.05$, solid $p < 0.01$).

(**n**) Distribution of Ecad::3xGFP at 24 hAPF in control wt (left) and ablated wt pupa (right, dashed red boxes) at 17 hAPF in the head and in the thorax tissue prior to folding onset. White box indicates region shown in center panel. In the left panel an equivalent region is shown in the control tissue. Orange arrows, average flow speed vectors from 21 hAPF to 24 hAPF measured by PIV (5 $\mu m.h^{-1}$, orange arrows in the bottom left). Note that tissue flows are strongly reduced except very close to the invagination position. Also note that in the ablated pupa, the cells are stretched along the AP axis (compare control, left panel with ablated, middle panel).

(**o**) Graph of apical neck depth (mean ± sem) in control wt pupae ($N = 10$ pupae) and in wt pupae where head and thorax tissue were ablated prior to folding (17 hAPF) ($N = 5$ pupae) or ablated at 21 hAPF ($N = 7$ pupae) as a function of developmental time. Horizontal boxes: *p*-values of Welch tests performed between experimental condition and the wt control at successive time points color coded according to experimental condition (white $p > 0.05$, striped $p < 0.05$, solid $p < 0.01$).

Source data are provided as a Source Data file.

Scale bars, 5 µm (g,k), 10 µm (a,b), 50 µm (n). Welch test, ns $p > 0.5$, *** $p < 0.001$ (c, d left and middle,i,j,l). *t*-test: ns $p > 0.05$, *** $p < 0.001$ (c,d right).

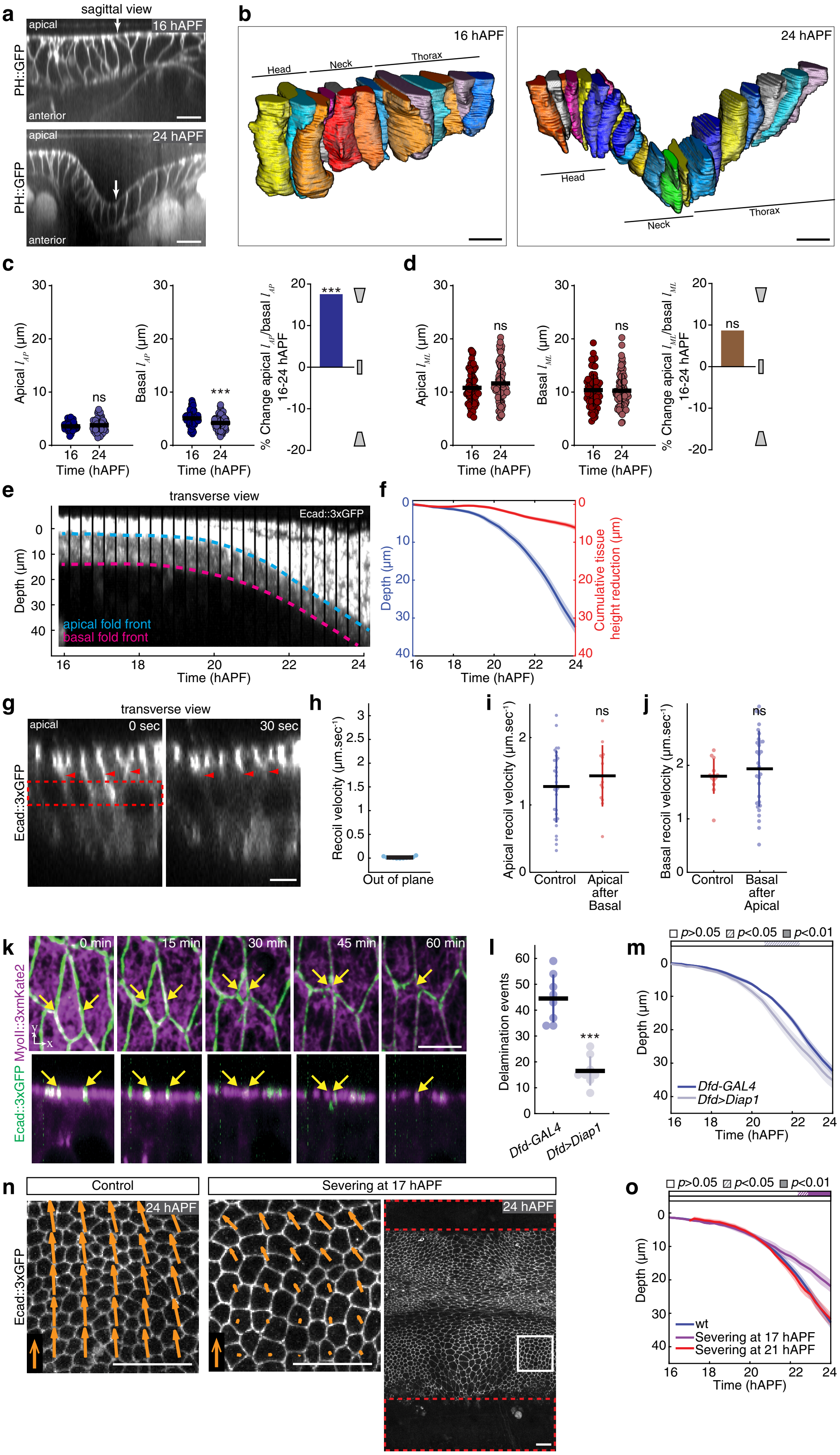

a
sagittal view
apical
16 hAPF
PH::GFP
anterior
apical
24 hAPF
PH::GFP
anterior
b
16 hAPF
Head
Neck
Thorax
24 hAPF
Head
Neck
Thorax
c
Apical l_{AP} (μm)
Basal l_{AP} (μm)
Time (hAPF)
ns
***
% Change apical l_{AP}/basal l_{AP} 16-24 hAPF
d
Apical l_{ML} (μm)
Basal l_{ML} (μm)
% Change apical l_{ML}/basal l_{ML} 16-24 hAPF
e
transverse view
Ecad::3xGFP
Depth (μm)
apical fold front
basal fold front
Time (hAPF)
f
Depth (μm)
Cumulative tissue height reduction (μm)
Time (hAPF)
g
transverse view
apical
0 sec
30 sec
Ecad::3xGFP
h
Recoil velocity (μm.sec^{-1})
Out of plane
i
Apical recoil velocity (μm.sec^{-1})
Control
Apical after Basal
j
Basal recoil velocity (μm.sec^{-1})
Control
Basal after Apical
k
0 min
15 min
30 min
45 min
60 min
Ecad::3xGFP MyoII::3xmKate2
l
Delamination events
Dfd-GAL4
Dfd>Diap1
m
p>0.05 p<0.05 p<0.01
Depth (μm)
Dfd-GAL4
Dfd>Diap1
Time (hAPF)
n
Control
Severing at 17 hAPF
24 hAPF
Ecad::3xGFP
o
p>0.05 p<0.05 p<0.01
Depth (μm)
wt
Severing at 17 hAPF
Severing at 21 hAPF
Time (hAPF)

Supplementary Fig. 7: Imaging with and without coverslip as well as Myosin and Ecad distributions as a function of curvature.

(**a**,**b**) Top view (top panels) and transverse view (bottom panels) images of the neck region labelled by Ecad::3xGFP at 18 hAPF of the same pupa imaged without coverslip using an air objective (a) and with a coverslip that locally flattened the tissue using an oil objective (b). Image acquisition settings (laser power and exposure time) are identical in the two conditions. As expected, the image quality is higher using an oil objective and a coverslip. Dashed line, midline position. Inset: enlargement of the square.

(**c,d**) Graphs of the normalized MyoII (c) and Ecad (d) signal (± sem) in the neck region along the ML axis as a function of the local tissue curvature in 21 hAPF pupae imaged with medial coverslip flattening (N = 10 pupae). While the curvature is spatially heterogeneous, the MyoII and Ecad signals remain homogeneous along the ML axis. ANOVA, $p = 0.9$.

Source data are provided as a Source Data file.

Scale bars, 50 µm.

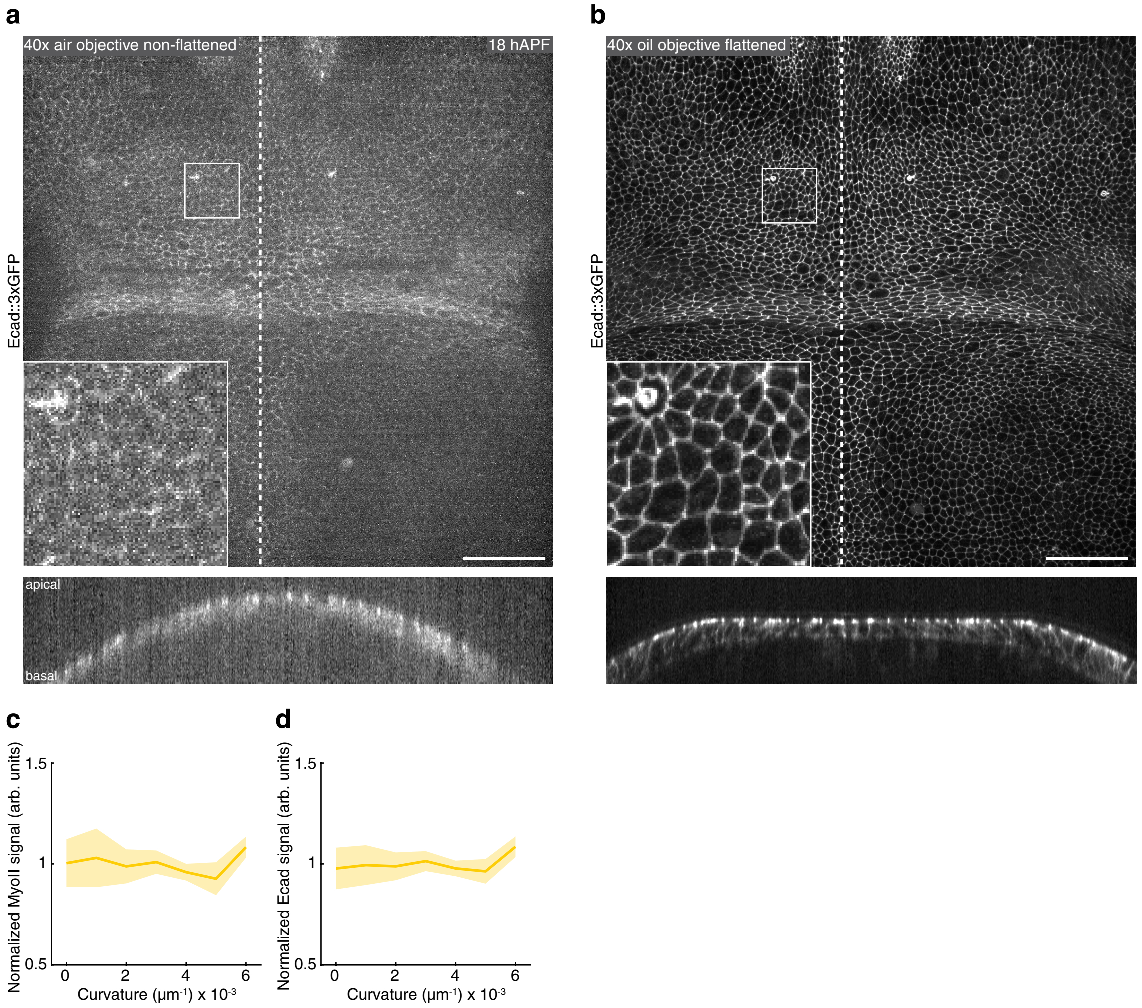
a
40x air objective non-flattened
18 hAPF
Ecad::3xGFP
apical
basal
b
40x oil objective flattened
Ecad::3xGFP
c
Normalized MyoII signal (arb. units)
Curvature (µm-1) x 10-3
d
Normalized Ecad signal (arb. units)
Curvature (µm-1) x 10-3

Supplementary Fig. 8: Apical recoil velocities as well as folding dynamics in wt and mutant conditions.

(**a**) Graphs of the ML apical initial recoil velocity (mean ± sd, averaged with a 2 h sliding window) after ablation in the neck region in control *Dfd-GAL4* (*N* = 125 pupae) and wt (*N* = 127 pupae) tissues, and in *Dfd>Rok*^{RNAi} (*N* = 57 pupae), *Dfd>MyoII*^{RNAi} (*N* = 48 pupae), *Dfd>Dfd*^{RNAi} (*N* = 106 pupae), *Dfd>Mbs*^{RNAi} (*N* = 61 pupae), *Dfd>Tollo*^{RNAi} (*N* = 90 pupae), *Dg* (*N* = 57 pupae) and *Dys* (*N* = 108 pupae) tissues. Horizontal box: *p-values* of Welch tests performed between experimental condition and either the *Dfd-GAl4* or wt control at successive time points (white $p > 0.05$, striped $p < 0.05$, solid $p < 0.01$).

(**b**) Graph of the apical fold front deepening speed and of the product of curvature and ML tension estimated by laser ablation in *Dfd>MyoII*^{RNAi}, *Dfd>Dfd*^{RNAi} and *Dys*. Datasets from Fig. 2e, Fig. 3a and Supplementary Fig. 8a were used (see corresponding figure legends for sample size). A line passing through the origin was fitted (R^2 values are indicated).

(**c**) Graph of the proportionality coefficients of deepening speed with respect to product of curvature times recoil velocity (± sd) for indicated genotypes. Datasets from Fig. 5e and Supplementary Fig. 8b were used (see corresponding figure legends for sample size). The slopes of the linear fit were extracted for each animal to calculate average slopes and the standard errors. Across all conditions, the slope average is 0.35 µm and its standard deviation is 0.06 µm.

Source data are provided as a Source Data file.

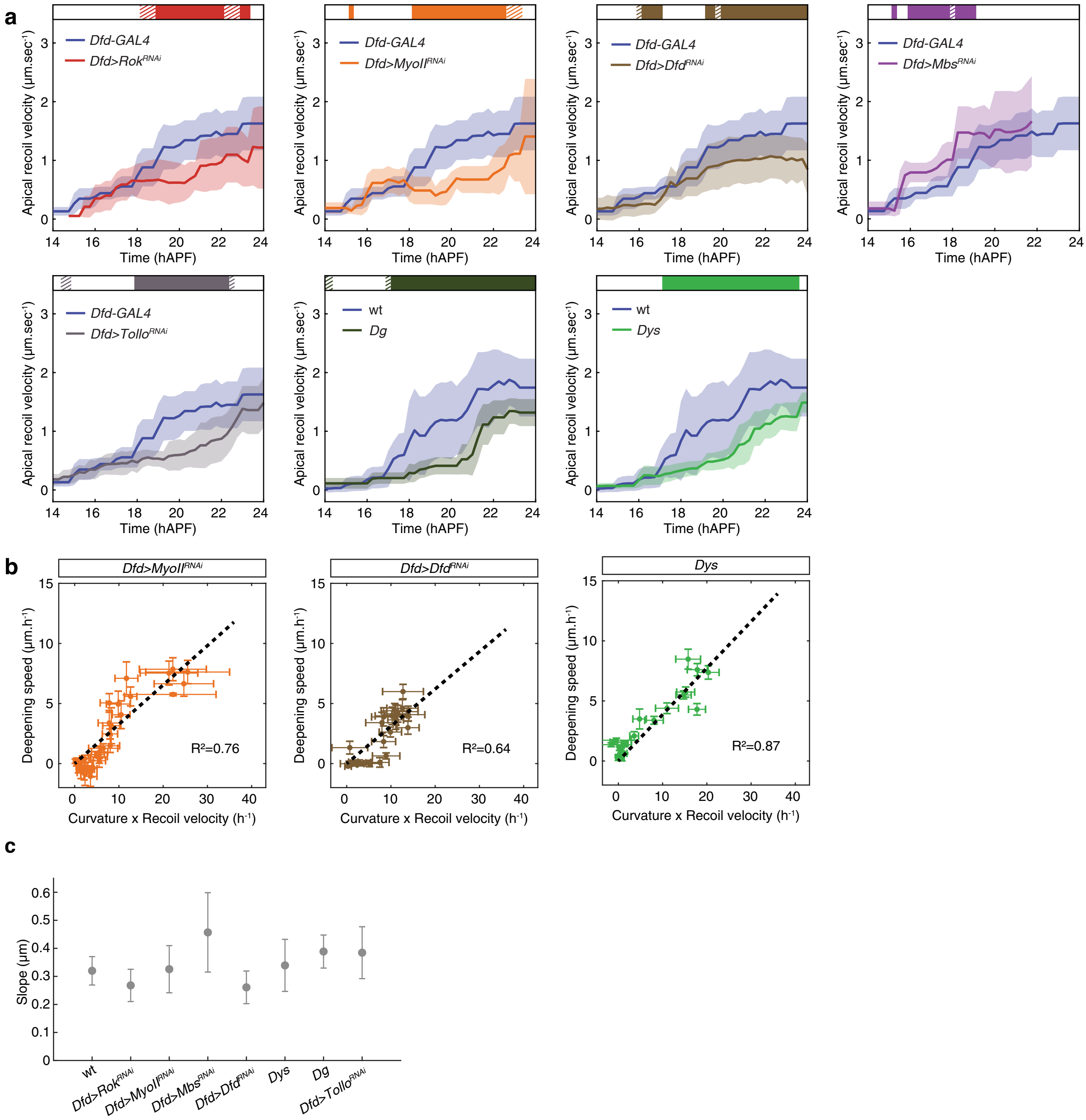

a
Dfd-GAL4
Dfd>Rok^RNAi
Dfd>MyoII^RNAi
Dfd>Dfd^RNAi
Dfd>Mbs^RNAi
Dfd>Tollo^RNAi
wt
Dg
Dys
Apical recoil velocity (µm.sec⁻¹)
Time (hAPF)
b
Dfd>MyoII^RNAi
Dfd>Dfd^RNAi
Dys
Deepening speed (µm.h⁻¹)
Curvature x Recoil velocity (h⁻¹)
R²=0.76
R²=0.64
R²=0.87
c
Slope (µm)
wt
Dfd>Rok^RNAi
Dfd>MyoII^RNAi
Dfd>Mbs^RNAi
Dfd>Dfd^RNAi
Dys
Dg
Dfd>Tollo^RNAi

Supplementary Fig. 9: Basal folding dynamics in wt tissues.

(**a**) Transverse view time-lapse images of the neck region labelled by Ecad::3xGFP at 18, 21 and 24 hAPF. Pink line, position of the basal fold front. Dashed line, midline position.

(**b**) Successive positions of the basal fold front color coded according to time (top), tissue curvature (middle) and the deepening speed (bottom) for an individual pupa. Dashed line, midline position.

(**c**) Graph of the basal fold front deepening speed versus curvature. Each point is the mean value (± sem) among 10 control animals for a given Position_{ML} at a given color-coded developmental time. For each developmental time, a line passing through the origin was fitted (R^2 values are indicated).

(**d**) Graph of the basal fold front deepening speed versus the product of curvature (mean ± sem) and ML basal initial recoil velocity after laser ablation in the neck region (mean ± sem) for a given Position_{ML} at a given color-coded developmental time. Datasets from Fig. 2d and Supplementary Fig. 9c were used (see corresponding figure legends for sample size). A line passing through the origin was fitted using average values ($R^2 = 0.81$).

Scale bars, 20 µm.

Source data are provided as a Source Data file.

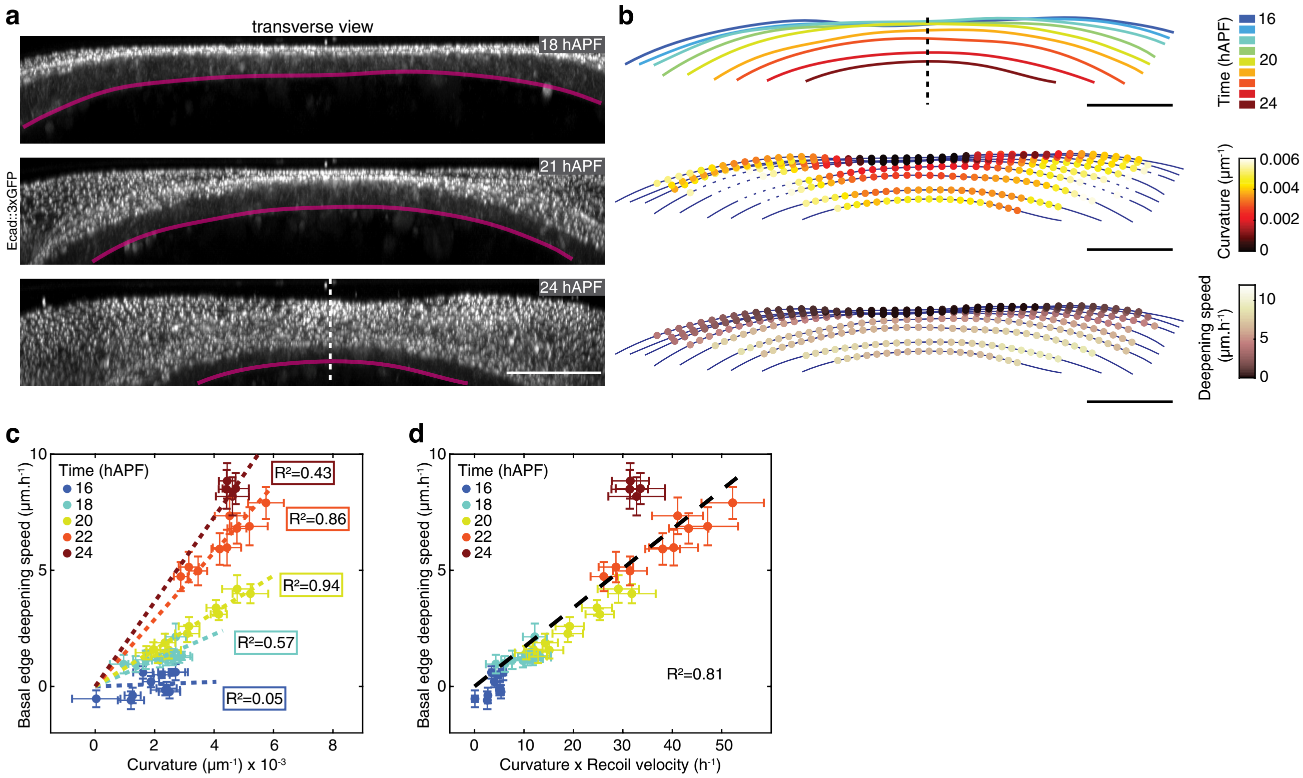

a
transverse view
18 hAPF
21 hAPF
24 hAPF
Ecad::3xGFP
b
Time (hAPF)
16
20
24
Curvature (µm⁻¹)
0.006
0.004
0.002
0
Deepening speed (µm.h⁻¹)
10
5
0
c
Basal edge deepening speed (µm.h⁻¹)
Time (hAPF)
16
18
20
22
24
R²=0.43
R²=0.86
R²=0.94
R²=0.57
R²=0.05
Curvature (µm⁻¹) x 10⁻³
d
Basal edge deepening speed (µm.h⁻¹)
Time (hAPF)
16
18
20
22
24
R²=0.81
Curvature x Recoil velocity (h⁻¹)

Supplementary Fig. 10: Basal recoil velocities as well as folding dynamics in wt and mutant conditions.

(**a**) Graphs of the ML basal initial recoil velocity (mean ± sd, averaged with a 2 h sliding window) after ablation in the neck region in control *Dfd-GAL4* (N = 125 pupae) and wt (N = 127 pupae) tissues, and in *Dfd>Rok*^{RNAi} (N = 57 pupae), *Dfd>MyoII*^{RNAi} (N = 48 pupae), *Dfd>Dfd*^{RNAi} (N = 106 pupae), *Dfd>Mbs*^{RNAi} (N = 61 pupae), *Dfd>Tollo*^{RNAi} (N = 90 pupae), *Dg* (N = 57 pupae) and *Dys* (N = 108 pupae) tissues. Horizontal box: *p-values* of Welch tests performed between experimental condition and either the *Dfd-GAl4* or wt control at successive time points (white $p > 0.05$, striped $p < 0.05$, solid $p < 0.01$).

(**b**) Graph of the basal fold front deepening speed (mean ± sem) and of the product of curvature and ML basal tension estimated by laser ablation (mean ± sem) for a given $\text{Position}_{\text{ML}}$ at a given developmental time for all tested experimental conditions. Datasets used are the ones in Fig. 5f for the tracking of the basal fold front and basal recoil velocity measurements from Supplementary Fig. 9 and Supplementary Fig. 10a (see corresponding figure legends for sample size). A line passing through the origin was fitted ($R^2 = 0.60$).

Source data are provided as a Source Data file.

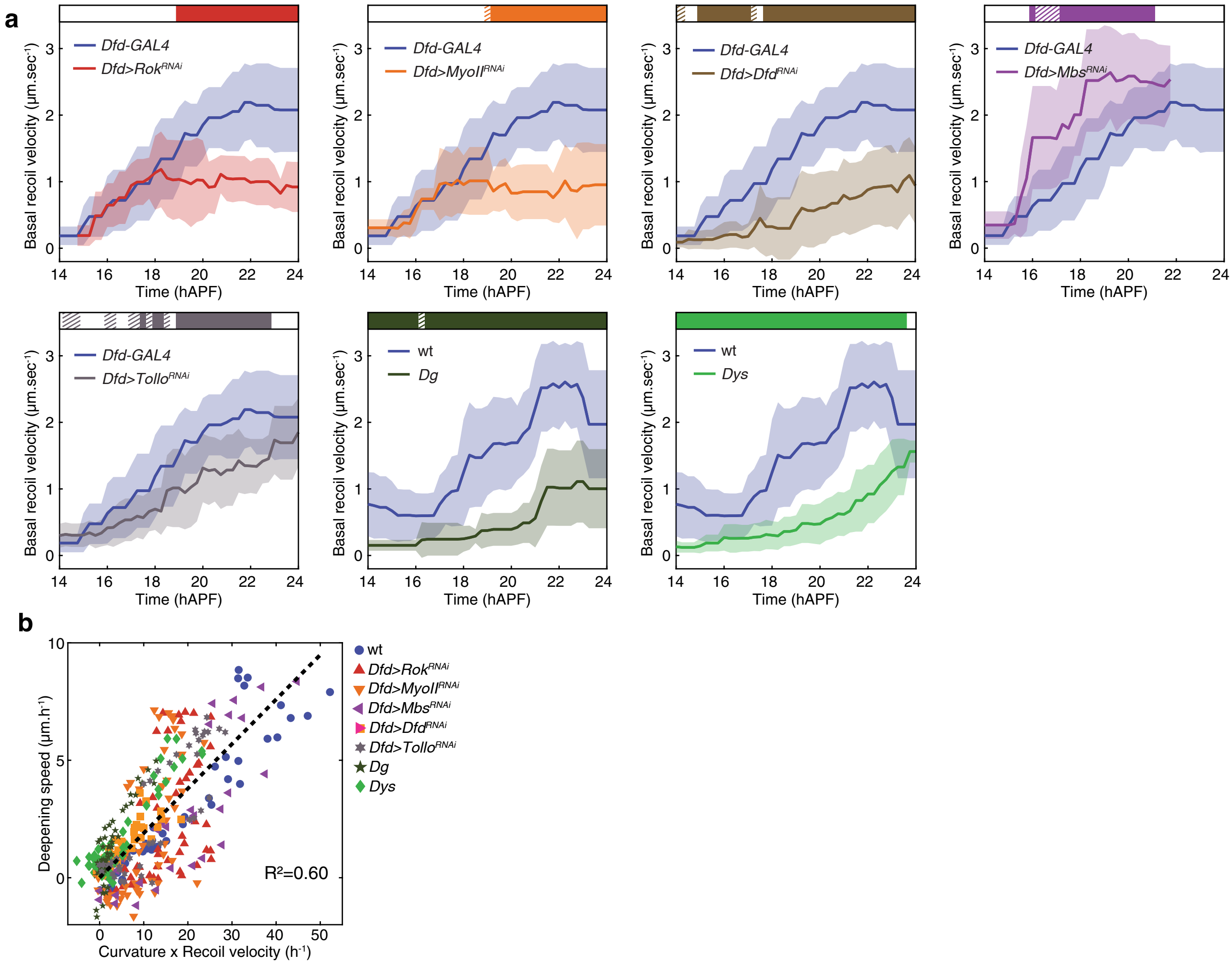
a
Dfd-GAL4
Dfd>Rok^RNAi
Dfd>MyoII^RNAi
Dfd>Dfd^RNAi
Dfd>Mbs^RNAi
Dfd>Tollo^RNAi
wt
Dg
Dys
Basal recoil velocity (µm.sec⁻¹)
Time (hAPF)
b
Deepening speed (µm.h⁻¹)
Curvature x Recoil velocity (h⁻¹)
R²=0.60
wt
Dfd>Rok^RNAi
Dfd>MyoII^RNAi
Dfd>Mbs^RNAi
Dfd>Dfd^RNAi
Dfd>Tollo^RNAi
Dg
Dys

Supplementary Fig. 11: Temporal evolution of neck curvature upon flattening.

(**a**) Graph of the ML apical initial recoil velocity (mean ± sd, averaged with a 2 h sliding window) after ablation in the neck region in pupae with (flattened, $N = 127$ pupae) and without (non-flattened, $N = 72$ pupae) coverslip flattening. Horizontal box: *p-values* of Welch tests performed between flattened and non-flattened pupae at successive time points (white $p > 0.05$, striped $p < 0.05$, solid $p < 0.01$).

(**b**) Graph of the ML basal initial recoil velocity (mean ± sd, averaged with a 2 h sliding window) after ablation in the neck region in pupae with (flattened, $N = 127$ pupae) and without (non-flattened, $N = 71$ pupae) coverslip flattening. Horizontal box: *p-values* of Welch tests performed between flattened and non-flattened pupae at successive time points (white $p > 0.05$, striped $p < 0.05$, solid $p < 0.01$).

(**c**) Graph of the proportionality coefficients of deepening speed with respect to product of curvature times recoil velocity in medially flattened, non-flattened and laterally flattened conditions (± sd). Datasets of Fig. 4d, and Fig. 5e,j were used (see corresponding figure legends for sample size). The slopes of the linear fit were extracted for each animal to calculate average slopes and the standard errors. Flattening affects both the curvature times tension and the velocity, but their relationship is unchanged (they remain proportional to each other with the same slope), indicating that the dissipation is unchanged, and more generally the morphogenetic mechanism remains the same.

(**d**) Graph of the curvature (blue, mean ± sem) and of the deepening speed (red, mean ± sem) of the apical fold front as a function of time for the flattened midline region in 10 control animals mounted using classical mounting with coverslip (flattening). Note that the curvature gradually increases in time, which is accompanied by an increase in the deepening speed.

(**e**) Color-coded evolution of the mean curvature of the apical fold front as a function of the $\text{Position}_{\text{ML}}$ in 10 control animals mounted with coverslip flattening. Note the propagation of curvature from lateral to the medial as the apical fold front deepens and the transition from heterogenous curvature before 19 hAPF to a homogenous curvature after 22 hAPF (outlined by black dashed arrows). White dashed line, midline position.

(**f**) Top view (top) and transverse view (bottom) images of Ecad::3xGFP labelling in the apical neck tissue at 16 hAPF prior to tissue severing ablation of each lateral side of the neck (red dashed boxes) and at 24 hAPF after ablation. A close up of the region outlined in orange is shown below the panel. White arrows indicate autofluorescent yolk granules. Note that these granules reside in the hemolymph underneath the epithelium prior to ablation (16 hAPF), but after ablation (24 hAPF), are near the most apical side of the tissue close to the cuticle which covers the epithelium (yellow arrow).

(**g**) Graph of the ML apical initial recoil velocities (mean ± sd) upon probe ablation in the medial neck region in pupae with coverslip flattening ($N = 8$) and in pupae with coverslip flattening after lateral side tissue severing ablations ($N = 10$). The probe ablations to measure recoil velocities were performed at 24 ± 0.5 hAPF, ~9h after the first lateral tissue severing ablations were performed to isolate the medial tissue region. Welch test $p = 0.54$.

(**h**) Graph of the ML basal initial recoil velocities (mean ± sd) upon probe ablation in the medial neck region in pupae with coverslip flattening ($N = 8$) and in pupae with coverslip flattening after lateral side tissue severing ablations ($N = 10$). The probe ablations to measure recoil velocities were performed at 24 ± 0.5 hAPF, ~9h after the first lateral tissue severing ablations were performed to isolate the medial tissue region. Welch test $p = 0.998$.

Source data are provided as a Source Data file.

Scale bars, 50 μm. Welch test, ns $p > 0.05$.

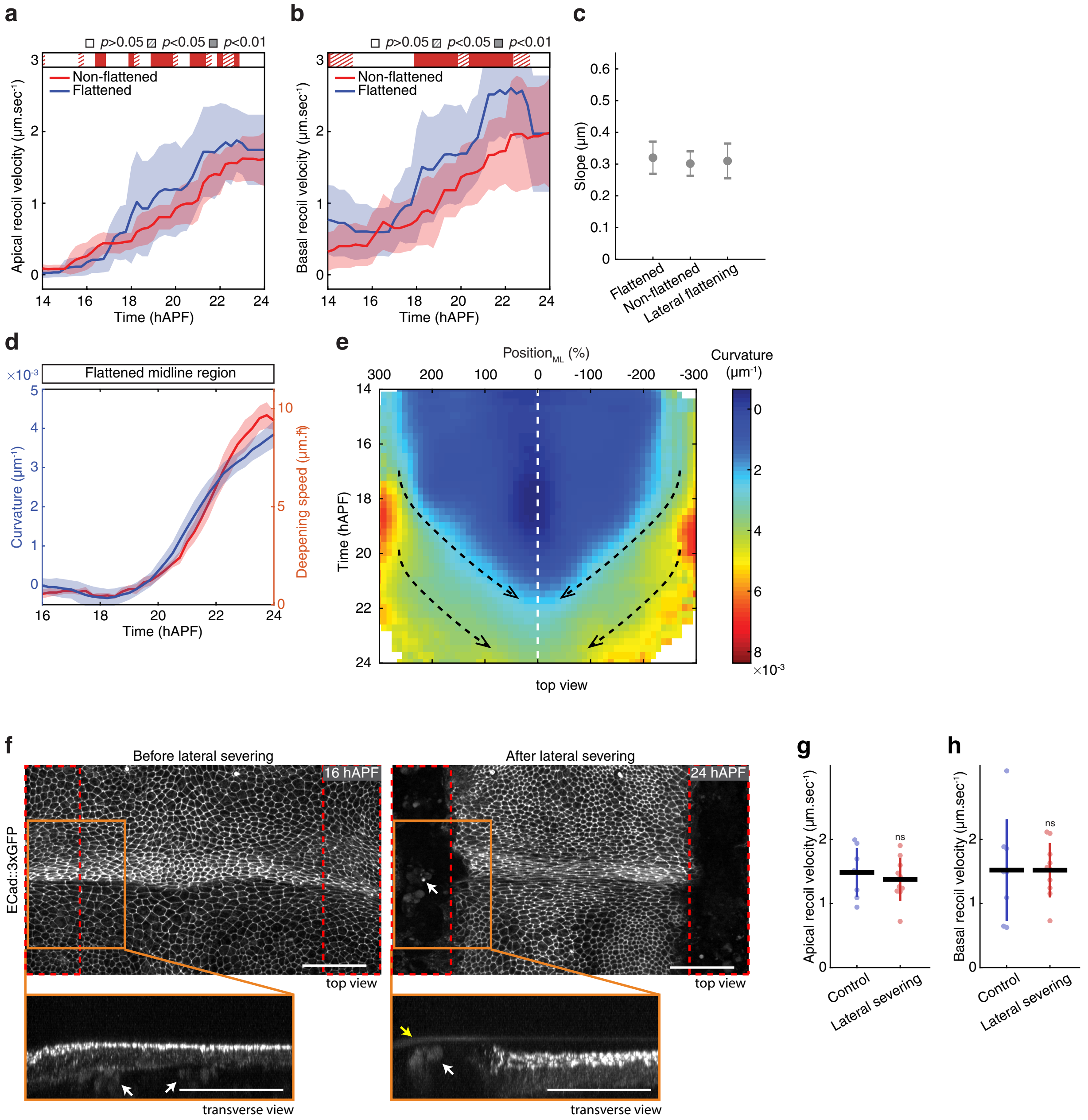

a
p>0.05 p<0.05 p<0.01
Non-flattened
Flattened
Apical recoil velocity (µm.sec⁻¹)
Time (hAPF)
b
Basal recoil velocity (µm.sec⁻¹)
c
Slope (µm)
Flattened
Non-flattened
Lateral flattening
d
Flattened midline region
Curvature (µm⁻¹)
Deepening speed (µm.h⁻¹)
e
Position_ML (%)
Curvature (µm⁻¹)
Time (hAPF)
top view
f
Before lateral severing
After lateral severing
16 hAPF
24 hAPF
ECad::3xGFP
top view
transverse view
g
Apical recoil velocity (µm.sec⁻¹)
Control
Lateral severing
ns
h
Basal recoil velocity (µm.sec⁻¹)

Supplementary Table 1. ***Drosophila*** **alleles and transgenes.**

Drosophila stock	*Reference or Source*
**Ubi-Ecad::GFP**	Ref [25,30,31]
**Ecad::3xGFP**	Ref [32]
**Ecad::3xmKate2**	Ref [32]
**utrABD::GFP**	Ref [33]
**MyoII::3xGFP**	Ref [32]
**MyoII::3xmKate2**	Ref [32]
**PH::GFP**	Ref [34]
**UAS-LifeAct::Ruby**	Bloomington Stock Center (#35545)
**Tollo::YFP**	Ref [35]
**Dg::GFP**	This study
**Dys::GFP**	Bloomington Stock Center (#59782)
**UAS-Diap1**	Bloomington Stock Center (#6657)
sqh^{RNAds}	Bloomington Stock Center (#33892)
Rok^{RNAds}	Bloomington Stock Center (#28797)
Mbs^{RNAds}	Bloomington Stock Center (#41625)
Dys^{RNAds}	Bloomington Stock Center (#55641)
Dfd^{RNAds}	Vienna Drosophila Resource Center (v50110)
Dg^{RNAds}	Vienna Drosophila Resource Center (107029)
$Tollo^{RNAds}$	Bloomington Stock Center (#28519)
$Tollo^{59}$	Ref [36]
$Tollo^{C5}$	Ref [37]
Dg^{086}	Ref [38]
Dg^{043}	Ref [38]
Dys^{E17}	Ref [39]
$Dys^{Exel6184}$	Ref [38]
**hs-flp**	Bloomington Stock Center (#8862)
**Act>CD2>Gal4, UAS-Caax:tBFP**	Ref [40]
**Act-GAL4**	Bloomington Stock Center (#3954)
**Ubi-GAL80**^{ts}	Bloomington Stock Center (#7017)
Dfd**-GAL4**	This study

Supplementary references

1. Martin, A. C., Kaschube, M. & Wieschaus, E. F. Pulsed contractions of an actin-myosin network drive apical constriction. *Nature* **457**, 495–499 (2009).
2. Chanet, S. *et al.* Actomyosin meshwork mechanosensing enables tissue shape to orient cell force. *Nat. Commun.* **8**, 1–13 (2017).
3. Sanchez-Corrales, Y. E., Blanchard, G. B. & Röper, K. Radially patterned cell behaviours during tube budding from an epithelium. *eLife* **7**, e35717 (2018).
4. Brooks, E. R., Islam, M. T., Anderson, K. V. & Zallen, J. A. Sonic hedgehog signaling directs patterned cell remodeling during cranial neural tube closure. *eLife* **9**, e60234 (2020).
5. Rauzi, M. *et al.* Embryo-scale tissue mechanics during Drosophila gastrulation movements. *Nat. Commun.* **6**, 8677 (2015).
6. Doubrovinski, K., Tchoufag, J. & Mandadapu, K. A simplified mechanism for anisotropic constriction in drosophila mesoderm. *Development* **145**, (2018).
7. Karzbrun, E. *et al.* Human neural tube morphogenesis in vitro by geometric constraints. *Nature* **599**, 268–272 (2021).
8. Pérez-González, C. *et al.* Mechanical compartmentalization of the intestinal organoid enables crypt folding and collective cell migration. *Nat. Cell Biol.* **23**, 745–757 (2021).
9. Yang, Q. *et al.* Cell fate coordinates mechano-osmotic forces in intestinal crypt formation. *Nat. Cell Biol.* **23**, (2021).
10. Krueger, D., Tardivo, P., Nguyen, C. & De Renzis, S. Downregulation of basal myosin- II is required for cell shape changes and tissue invagination. *EMBO J.* **37**, 1–16 (2018).
11. Sherrard, K., Robin, F., Lemaire, P. & Munro, E. Sequential Activation of Apical and Basolateral Contractility Drives Ascidian Endoderm Invagination. *Curr. Biol.* **20**, 1499–1510 (2010).
12. Sui, L. *et al.* Differential lateral and basal tension drive folding of Drosophila wing discs through two distinct mechanisms. *Nat. Commun.* **9**, 1–13 (2018).
13. Eritano, A. S. *et al.* Tissue-Scale Mechanical Coupling Reduces Morphogenetic Noise to Ensure Precision during Epithelial Folding. *Dev. Cell* **53**, 212-228.e12 (2020).
14. Urbano, J. M., Naylor, H. W., Scarpa, E., Muresan, L. & Sanson, B. Suppression of epithelial folding at actomyosin-enriched compartment boundaries downstream of Wingless signalling in Drosophila. *Development* **145**, dev155325 (2018).
15. Monier, B. *et al.* Apico-basal forces exerted by apoptotic cells drive epithelium folding. *Nature* **518**, 245–248 (2015).
16. Gracia, M. *et al.* Mechanical impact of epithelial−mesenchymal transition on epithelial morphogenesis in Drosophila. *Nat. Commun.* **10**, 1–17 (2019).
17. Nelson, C. M. On buckling morphogenesis. *J. Biomech. Eng.* **138**, 1–6 (2016).
18. Tozluoğlu, M. *et al.* Planar Differential Growth Rates Initiate Precise Fold Positions in Complex Epithelia. *Dev. Cell* **51**, 299-312.e4 (2019).
19. Shyer, A. E. *et al.* Villification: How the gut gets its villi. *Science.* **342**, 212–218 (2013).
20. Sidhaye, J. & Norden, C. Concerted action of neuroepithelial basal shrinkage and active epithelial migration ensures efficient optic cup morphogenesis. *eLife* **6**, e22689 (2017).
21. Heermann, S., Schütz, L., Lemke, S., Krieglstein, K. & Wittbrodt, J. Eye morphogenesis driven by epithelial flow into the optic cup facilitated by modulation of bone morphogenetic protein. *eLife* **2015**, e05216 (2015).
22. Ogura, Y., Wen, F. L., Sami, M. M., Shibata, T. & Hayashi, S. A Switch-like Activation Relay of EGFR-ERK Signaling Regulates a Wave of Cellular Contractility for Epithelial Invagination. *Dev. Cell* **46**, 162-172.e5 (2018).
23. Wang, Y. C. The origin and the mechanism of mechanical polarity during epithelial folding. *Semin. Cell Dev. Biol.* **120**, 94–107 (2021).
24. Lohmann, I., McGinnis, N., Bodmer, M. & McGinnis, W. The Drosophila Hox gene Deformed

sculpts head morphology via direct regulation of the apoptosis activator reaper. *Cell* **110**, 457–466 (2002).

25. Bosveld, F. *et al.* Mechanical Control of Morphogenesis by Fat/Dachsous/Four-Jointed Planar Cell Polarity Pathway. *Science.* **336**, 724–727 (2012).
26. Guirao, B. *et al.* Unified quantitative characterization of epithelial tissue development. *Elife* **4**, e08519 (2015).
27. Landau, L. D. & Lifshitz, E. M. (1986). Theory of Elasticity. Vol. 7 (3rd ed.). Butterworth–Heinemann.
28. Wettlaufer, J. S., Jackson, M. & Elbaum, M. A geometric model for anisotropic crystal growth. *J. Phys. A. Math. Gen.* **27**, 5957 (1994).
29. Gallet, F. La nature de la transition rugueuse : l'exemple des cristaux d'Hélium 4. PhD thesis, Université Pierre et Marie Curie - Paris 6, 1986).
30. Huang, J., Zhou, W., Dong, W., Watson, A. M. & Hong, Y. From the Cover: Directed, efficient, and versatile modifications of the Drosophila genome by genomic engineering. *Proc. Natl. Acad. Sci. U. S. A.* **106**, 8284–8289 (2009).
31. Oda, H., Tsukita, S. & Takeichi, M. Dynamic Behavior of the Cadherin-Based Cell–Cell adhesion system during Drosophila gastrulation. *Dev. Biol.* **450**, 435–450 (1998).
32. Pinheiro, D. *et al.* Transmission of cytokinesis forces via E-cadherin dilution and actomyosin flows. *Nature* **545**, 103–107 (2017).
33. Rauzi, M., Lenne, P. & Lecuit, T. Planar polarized actomyosin contractile flows control epithelial junction remodelling. *Nature* **468**, 1110–1114 (2010).
34. Herszterg, S., Leibfried, A., Bosveld, F., Martin, C. & Bellaiche, Y. Interplay between the Dividing Cell and Its Neighbors Regulates Adherens Junction Formation during Cytokinesis in Epithelial Tissue. *Dev. Cell* **24**, 256–270 (2013).
35. Paré, A. C. *et al.* A positional Toll receptor code directs convergent extension in Drosophila. *Nature* **515**, 523–527 (2014).
36. Yagi, Y., Nishida, Y. & Ip, Y. T. Functional analysis of Toll-related genes in Drosophila. *Dev. Growth Differ.* **52**, 771–783 (2010).
37. Akhouayri, I., Turc, C., Royet, J. & Charroux, B. Toll-8/Tollo Negatively Regulates Antimicrobial Response in the Drosophila Respiratory Epithelium. *PLOS Pathog.* **7**, e1002319 (2011).
38. Christoforou, C. P., Greer, C. E., Challoner, B. R., Charizanos, D. & Ray, R. P. The detached locus encodes Drosophila Dystrophin, which acts with other components of the Dystrophin Associated Protein Complex to influence intercellular signalling in developing wing veins. *Dev. Biol.* **313**, 519–532 (2008).
39. Campos, F. C. *et al.* Oriented basement membrane fibrils provide a memory for F-actin planar polarization via the Dystrophin-Dystroglycan complex during tissue elongation. *Development* **147**, dev186957 (2020).
40. López-Gay, J. M. *et al.* Apical stress fibers enable a scaling between cell mechanical response and area in epithelial tissue. *Science* **370**, eabb2169 (2020).
