# Supplementary material for: Homeotic compartment curvature and tension control spatiotemporal folding dynamics
Source: Nat Commun. 2023 Feb 3;14:594. doi: 10.1038/s41467-023-36305-6 (PMC9898526; doi:10.1038/s41467-023-36305-6)
Supplement: Supplementary file 3 — Description of Additional Supplementary Files [file 41467_2023_36305_MOESM3_ESM.pdf]

Description of Additional Supplementary Files

File Name: Supplementary Movie 1
Description: **Neck folding during pupal development.** Time-lapse 3D (top) movie of the dorsal side of Ecad::3xGFP pupa acquired in the neck region (blue) and the corresponding transverse view (bottom) movie between 14 and 24h40min hAPF. Scale bar, 50µm.

File Name: Supplementary Movie 2
Description: **Localization of Dfd, Tollo, MyoII, F-Actin and Dg during neck folding.**
(**a**) Images of Dfd (magenta) and Ecad::3xGFP (green) at 14, 18, 21 and 24 hAPF during neck fold formation. Dfd levels are constant throughout neck folding.

(**b, c**) Apical (b) and basal (c) MyoII::3xGFP (magenta) and F-Actin (green) localizations at 14, 18, 21 and 24 hAPF during neck fold formation. Apical MyoII accumulation is present in the neck cells at 14 hAPF and becomes more pronounced by 18 hAPF. From 21 hAPF onwards, MyoII gets increasingly polarized to accumulate in cell junctions (b). MyoII and F-actin are observed to form a network at the basal side of the neck at 14 hAPF. The anisotropy of this actomyosin network increases over time, becoming noticeably pronounced by 21 hAPF in the medial region of the neck (c).

(**d**) Images of Tollo::YFP (green) at 14, 18, 21 and 24 hAPF during neck fold formation. Tollo::YFP is enriched apically at neck cell junctions throughout neck invagination. From 21 hAPF onwards Tollo::YFP becomes also enriched in the lateral domains of the thorax.

(**e**) Images of Dg::GFP (green) and Dfd (magenta) at 14, 18, 21 and 24 hAPF during neck fold formation. Dg::GFP is localized in a basal network which becomes increasingly aligned in the ML direction within the neck region. At 24 hAPF, Dg::GFP accumulation in the neck is apparently lowered and a sagittal section is shown in inset for this time point to illustrate the presence of Dg::GFP at the basal side of Dfd positive neck cells. The two arrowheads indicate the positions between which the projected maximal sagittal sections were created.

Note that Movie 2 can also be opened as a stack in Fiji for easier visualization. Scale bar, 20µm

File Name: Supplementary Movie 3
Description: **Apical and basal laser ablation encompassing the presumptive neck region.** Time-lapse movies after laser ablation of the apical (top) and basal (bottom) neck tissue in pupae expressing utrABD::GFP. Dashed red boxes, ablated regions. Scale bar, 20µm.

File Name: Supplementary Movie 4
Description: **Dynamics of neck folding as function of neck region curvature.** Transverse view time-lapse movies of the neck region labelled by Ecad::3xGFP in pupae imaged with medial coverslip flattening (top), without coverslip flattening (middle), and with lateral coverslip flattening (bottom). The positions of the apical fold front are color-coded at 16, 18, 20, 22 and 24 hAPF on the last images. Scale bar, 50µm.

File Name: Supplementary Movie 5
Description: **Dynamics of neck folding after lateral tissue severing ablations in the presence or the absence of medial curvature.** Transverse view time-lapse movies of the neck region labelled by Ecad::3xGFP in a control pupa imaged with coverslip flattening (top) and in pupae with lateral tissue severing ablations and imaged without (middle) and with coverslip flattening (bottom). The positions of the apical fold front are color-coded at 16, 18, 20, 22 and 24 hAPF on the last images. Red dashed boxes, ablated regions. Scale bar, 50µm.
