# Supplementary material for: Homeotic compartment curvature and tension control spatiotemporal folding dynamics
Source: Nat Commun. 2023 Feb 3;14:594. doi: 10.1038/s41467-023-36305-6 (PMC9898526; doi:10.1038/s41467-023-36305-6)
Supplement: Supplementary file 9 — Reporting Summary [file 41467_2023_36305_MOESM9_ESM.pdf]

nature portfolio

Corresponding author(s): Floris Bosveld and Yohanns Bellaïche

Last updated by author(s): Jan 9, 2023

Reporting Summary

Nature Portfolio wishes to improve the reproducibility of the work that we publish. This form provides structure for consistency and transparency in reporting. For further information on Nature Portfolio policies, see our Editorial Policies and the Editorial Policy Checklist.

Statistics

For all statistical analyses, confirm that the following items are present in the figure legend, table legend, main text, or Methods section.

n/a	Confirmed	
☐	☒	The exact sample size (*n*) for each experimental group/condition, given as a discrete number and unit of measurement
☐	☒	A statement on whether measurements were taken from distinct samples or whether the same sample was measured repeatedly
☐	☒	The statistical test(s) used AND whether they are one- or two-sided<br>*Only common tests should be described solely by name; describe more complex techniques in the Methods section.*
☒	☐	A description of all covariates tested
☒	☐	A description of any assumptions or corrections, such as tests of normality and adjustment for multiple comparisons
☐	☒	A full description of the statistical parameters including central tendency (e.g. means) or other basic estimates (e.g. regression coefficient) AND variation (e.g. standard deviation) or associated estimates of uncertainty (e.g. confidence intervals)
☐	☒	For null hypothesis testing, the test statistic (e.g. *F*, *t*, *r*) with confidence intervals, effect sizes, degrees of freedom and *P* value noted<br>*Give P values as exact values whenever suitable.*
☒	☐	For Bayesian analysis, information on the choice of priors and Markov chain Monte Carlo settings
☒	☐	For hierarchical and complex designs, identification of the appropriate level for tests and full reporting of outcomes
☒	☐	Estimates of effect sizes (e.g. Cohen's *d*, Pearson's *r*), indicating how they were calculated

Our web collection on statistics for biologists contains articles on many of the points above.

Software and code

Policy information about availability of computer code

Data collection	Data collections were performed using microscope sotfware: Zeiss Zen (V2.3) and MetaMorph (V7.8.13.0).
Data analysis	Data Analyses and representations were performed using Matlab (V2018b), Imaris (x6 4.8.2), Excel (V2021), GraphPad PRism (V8.4.2) and Fiji (V1.53). All codes are available as https://doi.org/10.5281/zenodo.7521478

For manuscripts utilizing custom algorithms or software that are central to the research but not yet described in published literature, software must be made available to editors and reviewers. We strongly encourage code deposition in a community repository (e.g. GitHub). See the Nature Portfolio guidelines for submitting code & software for further information.

Data

Policy information about availability of data

All manuscripts must include a data availability statement. This statement should provide the following information, where applicable:

- Accession codes, unique identifiers, or web links for publicly available datasets
- A description of any restrictions on data availability
- For clinical datasets or third party data, please ensure that the statement adheres to our policy

All data are available from the corresponding authors upon reasonable request.

Human research participants

Policy information about studies involving human research participants and Sex and Gender in Research.

Reporting on sex and gender	NA
Population characteristics	NA
Recruitment	NA
Ethics oversight	NA

Note that full information on the approval of the study protocol must also be provided in the manuscript.

Field-specific reporting

Please select the one below that is the best fit for your research. If you are not sure, read the appropriate sections before making your selection.

☒ Life sciences ☐ Behavioural & social sciences ☐ Ecological, evolutionary & environmental sciences

For a reference copy of the document with all sections, see nature.com/documents/nr-reporting-summary-flat.pdf

Life sciences study design

All studies must disclose on these points even when the disclosure is negative.

Sample size	Sample sizes vary in each experiment. Animals were randomly selected within a given genotype for subsequent analyses. All samples sizes are reported in figure legends. The main limitations for higher sample size were microcospe availability and time taken to sample animals of the correct genotype and perform experiments.
Data exclusions	- Samples unproperly mounted for microscopy as well as the ones dying during live-imaging were excluded from subsequent analyses.<br>- As stated in the Method section: In the analysis of neck curvature: We exclude less than 2% of the total of the medial-lateral positions located on the edges of the tracked fold front and sampled in less than 5 animals or showing a curvature characterized by a sem higher than 0.002. This criteria were chosen based on the following technical limitations: the tracking of the neck front positions near the edge of the microscopic field is error-prone. We observed that the positions that were not tracked properly were characterized by a curvature sem among the animals stronger than 0.002µm-1. Based on this observation, we decided to filter out these positions. We checked that these excluded positions only corresponds to the edge of the field and that they correspond to only a small proportion of the dataset (less than 2 %)
Replication	All experiments were succesfully repeated at least twice.
Randomization	No randomization methods were used to determine how samples/animals were allocated. We randomly selected animals of the correct genotypes and developmental stage.
Blinding	No blind allocations during the experiments, nor when assessing the outcome. We did not use blind allocations since observables were quantified using unbiased and identical quantification methods in the differents experimental conditions.

Reporting for specific materials, systems and methods

We require information from authors about some types of materials, experimental systems and methods used in many studies. Here, indicate whether each material, system or method listed is relevant to your study. If you are not sure if a list item applies to your research, read the appropriate section before selecting a response.

Materials & experimental systems

n/a	Involved in the study	
☐	☒	Antibodies
☒	☐	Eukaryotic cell lines
☒	☐	Palaeontology and archaeology
☐	☒	Animals and other organisms
☒	☐	Clinical data
☒	☐	Dual use research of concern

Methods

n/a	Involved in the study	
☒	☐	ChIP-seq
☒	☐	Flow cytometry
☒	☐	MRI-based neuroimaging

Antibodies

Antibodies used	Primary antibodies used: mouse anti-Antp (DSHB, #8C11), rabbit anti-Dfd (1:100 dilution, gift from T. Kaufman). Secondary antibodies used: donkey anti-rabbit Cy5 (Interchim,cat.#711-175-152, 1:500), donkey anti-mouse Cy3 (Interchim, cat.# 711-165-152, 1:500).
Validation	Mouse Anti-Antp: https://pubmed.ncbi.nlm.nih.gov/2895027/<br>Rabbit and-Dfd: https://pubmed-ncbi-nlm-nih-gov/1971987/

Animals and other research organisms

Policy information about studies involving animals; ARRIVE guidelines recommended for reporting animal research, and Sex and Gender in Research

Laboratory animals	Drosophila Melanogaster. Strains are indicated in Supplementary Table S1. Female or male were used. Developmental times of each experiments is indicated in the figures or their legends.
Wild animals	No wild animals were used
Reporting on sex	female or male animals were used. Animal sex was only considered to perform genetic crossed and to follow relevant alleles or transgenes.
Field-collected samples	The study does not use field-collected samples
Ethics oversight	This study used invertebrates and does not require an ethical approval.

Note that full information on the approval of the study protocol must also be provided in the manuscript.
